# In ovo sodium butyrate administration differentially impacts growth performance, intestinal barrier function, immune response, and gut microbiota characteristics in low and high hatch-weight broilers

**DOI:** 10.1186/s40104-024-01122-4

**Published:** 2024-12-07

**Authors:** Muhammad Zeeshan Akram, Nadia Everaert, Aleksandra Dunisławska

**Affiliations:** 1 Department of Biosystems, Nutrition and Animal-Microbiota Ecosystems Laboratory, KU Leuven, Heverlee, 3001 Belgium; 2 Precision Livestock and Nutrition Unit, Gembloux Agro-Bio Tech, University of Liège, Gembloux, 5030 Belgium; 3 Department of Animal Biotechnology and Genetics, Faculty of Animal Breeding and Biology, Bydgoszcz University of Science and Technology, Bydgoszcz, 85-084 Poland

**Keywords:** Broiler production, Flock uniformity, Gut health, In ovo stimulation, Microbiome

## Abstract

**Background:**

Hatch weight (HW) affects broiler growth and low HW (LHW) often leads to suboptimal performance. Sodium butyrate (SB) has been shown to promote growth through enhanced intestinal health. This study investigated how broilers with different HW responded to in ovo SB injection and whether SB could enhance gut health and performance in LHW chicks. Ross 308 broiler eggs were injected on incubation d 12 with physiological saline (control) or SB at 0.1% (SB1), 0.3% (SB3), or 0.5% (SB5). Post-hatch, male chicks from each treatment were categorized as high HW (HHW) or LHW and assigned to 8 groups in a 4 × 2 factorial design. Production parameters were recorded periodically. Intestinal weight, length, and gene expression related to gut barrier function and immune response were examined on d 14 and 42. Cecal microbiota dynamics and predicted functionality were analyzed using 16S rRNA gene sequencing.

**Results:**

SB treatments did not affect hatchability. HHW-control group exhibited consistently better weight gain and FCR than LHW-control group. SB dose-dependently influenced performance and gut health in both HW categories, with greater effects in LHW broilers at 0.3%. LHW-SB3 group attained highest body weight on d 42, exceeding controls but not significantly differing from HHW-SB3 group. LHW-SB3 group showed upregulation of gut-barrier genes *CLDN1* in ileum, *TJP1* in jejunum and anti-inflammatory cytokine *IL-10* in both jejunum and ileum on d 14. Additionally, LHW-SB3 group upregulated mucin-producing *MUC6* gene in ileum, while HHW-SB5 group increased pro-inflammatory *IL-12p40* cytokine in caecum on d 42. LHW-SB3 group demonstrated shorter relative intestinal lengths, while HHW-SB5 had longer lengths. HHW-control group had higher bacterial diversity and growth-promoting bacteria while LHW-control group harbored the potential pathogen *Helicobacter*. SB reshaped gut microbiota biodiversity, composition, and predicted metabolic pathways in both HW categories. The LHW-SB3 group exhibited highest alpha diversity on d 14 and most beneficial bacteria at all timepoints. HHW-SB5 group presented increased pathogenic *Escherichia-Shigella* and *Campylobacter* on d 42.

**Conclusions:**

HW significantly affects subsequent performance and SB has differential effects based on HW. LHW chicks benefited more from 0.3% SB, showing improvements in growth, intestinal development, health, and gut microbiota characteristics.

**Supplementary Information:**

The online version contains supplementary material available at 10.1186/s40104-024-01122-4.

## Background

The global demand for poultry meat continues to increase due to the growing human population, placing substantial pressure on broiler producers to enhance production efficiency [1]. Achieving uniform growth and higher slaughter weights in broiler chickens are essential objectives for meeting this demand [2]. Despite advances in genetic selection focused on production traits, significant variations in body weight (BW) within broiler flocks yet persist, often resulting in flocks being divided into two extreme low and high-weight categories [3]. Low-weight chicks frequently fail to reach their genetic potential, which leads to suboptimal performance, health issues, welfare concerns, and management challenges [4].

Numerous studies have compared low and high-weight chickens at slaughter, but few have considered hatch weight (HW) as a criterion for differentiating between low and high-performing broilers. HW has been identified as a strong predictor of subsequent growth performance in broilers [5]. Low HW (LHW) chicks typically exhibit slower growth rates, reduced slaughter weights, and suboptimal feed efficiency compared to their high HW (HHW) counterparts [6]. Recent research links low-weight incidences in chickens to intestinal dysfunction, increased permeability, and downregulation of tight junction proteins in the jejunum and ileum [7, 8]. Additionally, low-weight chickens often harbor imbalanced microbiota with pathogenic potential, leading to dysbiosis and increased intestinal inflammation [3], as evidenced by elevated pro-inflammatory cytokine expression [8].

Butyric acid, a short-chain fatty acid (SCFA), has gained attention as a feed additive in poultry production due to its potential benefits on gut health, growth performance, and immune modulation [9]. Butyrate accelerates gut epithelial cell proliferation, improves mucosal morphology, and enhances weight gain and carcass characteristics in chickens [10, 11]. It also exerts immunomodulatory effects by inducing host defense peptides, modulating cytokine expression, and increasing IgG and IgA levels [12–14]. Furthermore, it has been shown to reduce the incidence of intestinal inflammation, thereby contributing positively to overall gut health [15]. Butyrate supports beneficial microbiota growth by lowering the intestinal pH, creating an unfavorable environment for pathogenic bacteria [16]. As a result, the digestion and absorption of nutrients are enhanced, effectively improving the growth performance of animals [17].

Early-life interventions in broiler chickens, particularly during the 21 d incubation period, can significantly impact their long-term health and performance. The small intestine initiates differentiation and morphological changes around embryonic d 14 (ED14), while immune system development begins around ED10, with T cells and B cells developing around ED12 [18, 19]. The microbiota in the egg, especially within the yolk sac and amniotic fluid, shifts throughout embryonic development, indicating that the native bacteria present in the egg may play a role in development [20]. Given this developmental timeline, in ovo butyrate stimulation on incubation d 12 can shape gastrointestinal tract (GIT) related parameters, which may have lasting effects on overall broiler performance throughout the production cycle. Previous studies have shown that in ovo administration of bioactive substances on d 12 of incubation can effectively modulate the gut microbiota and immune response [21].

Previous studies have demonstrated the effects of butyrate on broiler chickens’ health status and intestinal response with normal HW. However, it remains unknown whether LHW chicks respond similarly to butyrate as their normal HW counterparts or whether butyrate can mitigate delayed growth effects in LHW chicks. LHW chickens typically exhibit slower growth rates and suboptimal feed efficiency compared to their HHW counterparts [6]. These differences could be linked to variations in intestinal development, gut microbiota composition, and immune function [3, 7]. Given these differences, it is plausible that LHW chickens may respond differently to in ovo butyrate administration. This study is the first to investigate the effects of HW on growth performance, intestinal development and function, and microbiota composition in broilers and how these effects are influenced by in ovo sodium butyrate (SB) injection. We hypothesized that in ovo SB administration will improve performance, support intestinal barrier function, regulate the immune response, and modulate the gut microbiota composition and function more effectively in LHW chickens than in their HHW counterparts.

## Methodology

### Eggs and in ovo injection

Ross 308 breeder eggs with an average weight of 66.5 ± 1.93 g originating from a 40-week-old breeding flock were obtained from a commercial hatchery (Drobex-Agro, Solec Kujawski, Poland). All the eggs were incubated under standard conditions (37.8 °C and 60% relative humidity). On the 12^th^ days of incubation after candling, the eggs were randomly divided into 4 treatment groups (*n* = 300 eggs/group). Eggs were then injected into the air chamber with 0.2 mL of physiological saline (0.9% sodium chloride; Fresenius Kabi, Warsaw, Poland) or 1 of 3 doses of SB (molecular weight: 110.09 g; Merck Life Science, Warsaw, Poland). The treatment groups were as follows: (1) control (0.9% NaCl), (2) 0.1% SB (SB1), (3) 0.3% SB (SB3), and (4) 0.5% SB (SB5). The in ovo injection procedure was performed following the method described by Dunisławska et al. [22], and the eggs were incubated for 21 d.

### Post-hatch chick selection and management

**Fig. 1:**
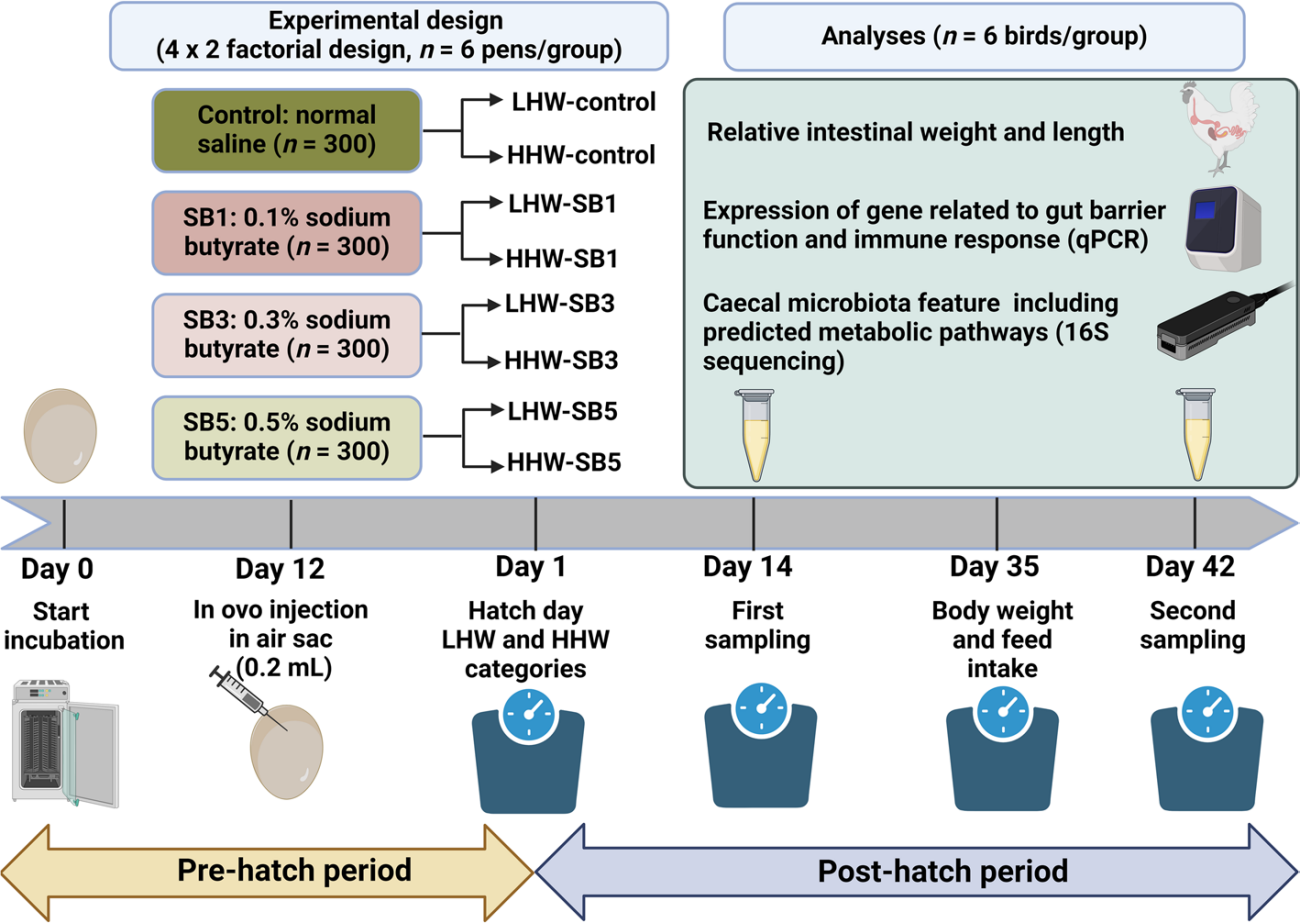
Flow chart illustrating the study design, timeline, and parameters investigated. This diagram was created using Biorender.com

At hatch, the hatchability of each in ovo SB treatment was recorded. The weights of the male chicks were recorded, and the chicks were categorized based on their HW. In each in ovo treatment group, chicks were divided into low and high HW groups, with 72 chicks per group, resulting in 576 chicks who continued in the experiment. LHW chicks had a BW of 45.6 ± 2.30 g, while HHW chicks weighed 55.1 ± 2.83 g. This created a 4 (SB) × 2 (HW) factorial arrangement, with 6 replicate pens per group and 12 chicks per pen (Fig. 1). The pens contained wheat straw litter as bedding material and had a single feeder and drinker. Uniform rearing conditions with appropriate ventilation, litter management, lighting programs, and stocking densities were provided as recommended by the Aviagen Ross 308 guidelines. The temperature of the barn was initially set at 33 °C, which decreased by approximately 0.5 °C daily until it reached 21.5 °C on d 21, after which it remained constant. Broilers had ad-libitum access to feed and water and had diets formulated for starter (1–14 d), grower (15–35 d), and finisher (36–42 d) phases; these diets contained 23.0%, 21.5%, and 19.5% crude protein and 3,000, 3,100, and 3,200 kcal/kg metabolizable energy, respectively.

### Growth performance parameters and sample collection

Individual BW and feed intake per pen were recorded at the end of each diet phase to calculate the average daily gain (ADG), average daily feed intake (ADFI), and feed conversion ratio (FCR). ADFI and FCR were adjusted for leftover feed and bird mortality. On d 14 and 42, 6 birds per group were stunned by percussive blows to the head and then decapitation in accordance with European Commission Council Regulation No. 1099/2009 of 24 September 2009 on the protection of animals at the time of killing [23]. After sacrifice, gut development parameters, including the weight and length of the small intestine segments (duodenum, jejunum, and ileum) and the cecum, were measured. The relative organ weights and intestine lengths are expressed as g/100 g BW and cm/100 g BW, respectively. Mucosa scrapings from the jejunum, ileum, and cecum were collected in RNA stabilizing buffer (fix RNA, EURx, Gdańsk, Poland) and stored at −80 °C until RNA extraction. The cecal digesta was collected, placed on dry ice, and stored at −80 °C until DNA extraction for microbiota analysis.

### Gene expression in the intestinal mucosa

#### RNA extraction

RNA was extracted from 100 mg of intestinal mucosal scrapings using an RNA extraction solution (EURx, Gdańsk, Poland) and a TissueRuptor homogenizer (Qiagen, Germany). The homogenate was centrifuged with 0.2 mL of chloroform (Chempur, Poland), and RNA was purified using a universal RNA purification kit (EURx, Poland). RNA quantity and quality were assessed using a NanoDrop 2000 spectrophotometer (Thermo Scientific, USA), and RNA integrity was examined on a 2% agarose gel.

#### Quantitative real-time PCR (qPCR)

Gene expression of gut barrier components (*CLDN1*, *TJP1*, and *MUC6*) and immune-related cytokines (*IL-1β*, *IL-12p40*, and *IL-10*) was quantified via qPCR, with *ACTB* and *G6PDH* serving as reference genes (Table S1). The RNA was reverse transcribed into cDNA using a SMART First Strand cDNA Synthesis Kit (EURx, Poland). Each sample was analyzed in duplicate with a LightCycler 480 System (Roche Diagnostics, Basel, Switzerland). The qPCR reactions were conducted in a 12.5-µL total volume and included 6.25 µL of SYBR Green I dye (EURx, Gdańsk, Poland), 1 µmol/L each of the forward and reverse primers, and 140 ng of cDNA. The qPCR protocol involved an initial denaturation step at 95 °C for 15 min, followed by 40 cycles of amplification (95 °C for 15 s, 58 °C for 20 s, and 72 °C for 20 s), and a melting curve analysis. Relative gene expression was calculated using the ΔΔCt method and quantified with the 2^−ΔΔCt^ formula as described by Livak and Schmittgen [24].

### Microbiota analysis

#### DNA extraction

DNA was isolated from approximately 150 mg of cecal digesta using a Stool DNA Purification Kit (EURx, Poland) following the manufacturer’s instructions. DNA quantity and quality were assessed as described in the RNA extraction section. The DNA samples were stored at −80 °C until further analysis.

#### ONT MinION (16S, V1−V9) library preparation and sequencing

DNA was prepared for prokaryotic metagenome sequencing using a 16S barcoding kit (SQK-16S024, Oxford Nanopore Technologies, Oxford, UK), with PCR amplification of the full hypervariable region (V1–V9) using universal 16S forward (27F): 5′-AGAGTTTGATCMTGGCTCAG-3′ and reverse (1492R): 5′-CGGTTACCTTGTTACGACTT-3′ primers. The obtained amplicons were purified with 30 µL of AMPure XP beads (Beckman Coulter, USA) and eluted in 10 µL of 10 mmol/L Tris-HCl buffer to a final library concentration of 100 fmol. The generated sequencing libraries were sequenced on a MinION Flow Cell (FLO-MIN-106, Oxford Nanopore Technologies, Oxford, UK) for 48 h, and the obtained data were processed into FASTQ files using the Ont-guppy-cpu basecaller (v 6.4.6, Oxford Nanopore Technologies, Oxford, UK) in super accurate mode.

#### Bioinformatics workflow

Raw reads underwent initial processing, which included demultiplexing, trimming, and quality-based filtering, using an Ont-guppy-cpu barcoder (v 6.5.7) and Nanofilt (v 2.8.0) software. Filtered FASTQ files were subsequently imported into QIIME 2 (v 2023.9) for downstream analysis. Dereplication of sequences was performed using vsearch [25], followed by de novo clustering of operational taxonomic units (OTUs) with an identity threshold of 85%. Taxonomic classification of clustered OTUs was performed against the SILVA database (release 138) using QIIME 2-vsearch with an 85% identity threshold. Alpha diversity metrics, calculated using the Shannon and Simpson indexes, were calculated after rarefying the OTU table to the minimum sample depth in R (v4.2.3). Differences in alpha diversity metrics between groups were assessed using two-way ANOVA. The Bray‒Curtis distance was used for the comparison of beta diversity data among groups via R and was visualized through principal coordinate analysis (PCoA). The significance of multivariate effects on beta diversity was tested using nonparametric permutational multivariate analysis of variance (PERMANOVA). Significant differences in the microbial communities were detected with linear discriminant analysis (LDA) effect size (LEfSe) in R with a minimum LDA threshold of 3.0. The obtained *P* values were further subjected to a false discovery rate (FDR) analysis using the Benjamin–Hochberg method. Phylogenetic investigation of the communities by reconstruction of unobserved states (PICRUSt2) was used to predict the functional capabilities of the microbial communities in the different groups using two-way ANOVA (*P* < 0.05), using the MetaCyc metabolic pathway database as a reference [26].

### Statistical analysis

The normality of the data was assessed through the Shapiro‒Wilk test in R. One-way ANOVA was applied to the hatching data. Two-way ANOVA was used to determine the significant effects of HW, SB, or their interaction on growth performance, intestinal weight and length, or gene expression. The means were separated by Tukey’s multiple comparison test, and the significance level was considered at *P* < 0.05. Heatmaps were generated in R using the pheatmap package (v 1.0.12) to visualize sample variability, with predicted microbial metabolic pathway values scaled by row. Heatmaps were based on Pearson’s correlation distance and ward clustering method for two-way hierarchical clustering analysis. Correlations between the most abundant bacterial genera and between bacterial genera and metabolic pathways were assessed by Pearson’s correlation analysis.

## Results

### Hatchability and growth performance

Hatchability was not affected by in ovo SB treatments (*P* > 0.05; Fig. S1). At hatching, BW differed significantly between the LHW and HHW categories (*P* < 0.001; Table 1), with HHW chicks having higher BW. A significant interaction effect between HW and SB on BW was observed on d 35 and 42 (*P* = 0.029 and *P* = 0.045, respectively). On d 35, the LHW-SB3 group had greater BW than both the LHW-control and HHW-SB5 groups. By d 42, the LHW-SB3 chicks had greater BW than LHW and HHW control groups but did not differ significantly from the HHW-SB3 and LHW-SB1 groups. ADG showed an interaction effect between HW and SB (*P* < 0.001), with LHW-SB1 having greater ADG during 1–14 d, while LHW-SB3 demonstrated greater ADG in all subsequent growth stages. The HHW-control group had greater ADG than the LHW-control group throughout the study. The ADFI was affected by the main effect of SB only during 15–35 d, with SB5-treated chicks showing higher feed intake and SB1-treated chicks showing lower intake (*P* = 0.029; Table 2). The FCR exhibited a significant interaction between HW and SB on 15–35 d and 36–42 d (*P* = 0.034 and *P* < 0.001, respectively). The LHW-SB3 group had the lowest FCR values for both periods, while the HHW-SB5 during 15–35 d and LHW-SB5 during 36–42 d showed the highest FCR values. Regardless of HW, the main effect of SB revealed that SB3-treated chicks were most feed efficient during 15–35 d, 36–42 d, and the overall 1–42 d period, while SB5-treated chicks were least efficient (*P* < 0.05).

**Table 1:** Effects of in ovo sodium butyrate administration on the body weight and average daily gain of broiler chickens with different hatch weights

Item^1^		Body weight, g				Average daily gain, g/d			
		1 d	14 d	35 d	42 d	0–14 d	15–35 d	36–42 d	0–42 d
HW	SB								
HHW	Control	55.18	399	1,917^ab^	2,418^b^	24.85^bc^	71.96^bc^	72.73^c^	56.87^bc^
	SB1	55.08	411	1,891^ab^	2,365^bc^	25.78^bc^	70.45^c^	67.45^d^	54.93^cd^
	SB3	55.06	397	2,028^ab^	2,571^ab^	24.74^c^	76.15^a^	75.92^bc^	59.42^b^
	SB5	55.20	335	1,668^c^	2,282^bc^	20.34^d^	63.50^d^	84.60^a^	54.91^cd^
LHW	Control	45.12	377	1,723^bc^	2,201^c^	24.01^c^	64.80^d^	67.87^d^	51.80^d^
	SB1	45.48	441	1,944^ab^	2,499^ab^	28.55^a^	71.26^bc^	79.05^b^	59.23^b^
	SB3	46.05	416	2,050^a^	2,651^a^	26.77^ab^	77.48^a^	87.01^a^	63.36^a^
	SB5	45.78	379	1,912^ab^	2,342^bc^	24.10^c^	72.67^b^	61.93^e^	53.68^cd^
SD		2.301	54.5	221.3	238.7	3.112	4.831	8.434	3.773
Main effects									
HW									
HHW		55.13^a^	386	1,876	2,409	23.93^b^	70.52^b^	75.18^a^	56.53
LHW		45.61^b^	403	1,907	2,423	25.86^a^	71.55^a^	73.97^b^	57.02
SB									
Control		50.15	388	1,820^b^	2,310^c^	24.43^c^	68.38^c^	70.30^c^	54.34^c^
SB1		50.28	426	1,918^ab^	2,432^b^	27.17^a^	70.86^b^	73.25^b^	57.08^b^
SB3		50.56	408	2,039^a^	2,611^a^	25.76^b^	76.82^a^	81.47^a^	61.39^a^
SB5		50.49	357	1,790^b^	2,312^c^	22.22^d^	68.09^c^	73.27^b^	54.30^c^
*P* value									
HW		< 0.001	0.087	0.576	0.668	0.079	0.029	0.027	0.256
SB		0.4191	0.108	< 0.001	0.025	0.023	< 0.001	< 0.001	< 0.001
HW × SB		0.419	0.061	0.029	0.045	< 0.001	< 0.001	< 0.001	< 0.001

^1^*HW *Hatch weight, *SB *Sodium butyrate inclusion level. Control: HHW or LHW chicks from eggs injected with 0.2 mL of 0.9% NaCl. SB1, SB3, SB5: HHW or LHW chicks from eggs injected with 0.2 mL of 0.1%, 0.3%, or 0.5% SB, respectively

Body weight was recorded from individual birds, while a pen was considered the experimental unit for average daily gain (*n* = 6 pens/group). The data are presented as the mean and pooled standard deviation (SD)

^a–d^Values with different superscripts in a column indicate statistical significance at *P* < 0.05 (two-way ANOVA followed by Tukey’s HSD test)

**Table 2:** Effects of in ovo sodium butyrate administration on the feed intake and feed conversion ratio of broiler chickens with different hatch weights

Item^1^		Average daily feed intake, g/bird/d				Feed conversion ratio			
		0–14 d	15–35 d	36–42 d	0–42 d	0–14 d	15–35 d	36–42 d	0–42 d
HW	SB								
HHW	Control	35.3	134.9	178.3	113.7	1.46	1.89^ab^	2.51^bc^	1.96
	SB1	34.9	132.9	180.9	113.6	1.39	1.92^ab^	2.70^ab^	2.00
	SB3	33.9	134.1	177.6	112.8	1.41	1.74^b^	2.35^cd^	1.83
	SB5	33.4	142.1	184.2	117.7	1.68	2.27^a^	2.13^d^	2.11
LHW	Control	34.2	133.1	181.4	113.5	1.47	2.10^a^	2.69^ab^	2.12
	SB1	35.2	132.9	181.6	114.0	1.27	1.89^ab^	2.32^cd^	1.86
	SB3	33.4	133.5	182.6	113.8	1.28	1.74^b^	2.12^d^	1.75
	SB5	33.8	136.2	177.8	113.5	1.44	1.89^ab^	2.92^a^	2.04
SD		3.27	4.17	3.72	5.07	0.177	0.210	0.301	0.174
Main effects									
HW									
HHW		34.4	135.9	180.2	114.4	1.49	1.96	2.42	1.98
LHW		34.2	133.9	180.9	113.7	1.37	1.91	2.51	1.94
SB									
Control		34.8	133.9^b^	179.9	113.6	1.46	1.99^a^	2.60^a^	2.04^b^
SB1		35.1	132.9^c^	181.3	113.7	1.33	1.91^ab^	2.51^a^	1.93^bc^
SB3		33.6	133.8^b^	180.1	113.2	1.35	1.74^b^	2.24^b^	1.79^c^
SB5		33.6	139.1^a^	181.0	115.6	1.56	2.08^a^	2.53^a^	2.08^a^
*P* value									
HW		0.882	0.165	0.668	0.770	0.069	0.448	0.181	0.537
SB		0.840	0.029	0.865	0.901	0.059	0.010	0.005	0.013
HW × SB		0.982	0.491	0.064	0.887	0.573	0.034	< 0.001	0.324

^1^*HW *Hatch weight, *SB *Sodium butyrate inclusion level. Control: HHW or LHW chicks from eggs injected with 0.2 mL of 0.9% NaCl. SB1, SB3, SB5: HHW or LHW chicks from eggs injected with 0.2 mL of 0.1%, 0.3%, or 0.5% SB, respectively

The data are presented as the mean and pooled standard deviation (SD) (*n* = 6 pens/group)

^a–d^Values with different superscripts in a column indicate statistical significance at *P* < 0.05 (two-way ANOVA followed by Tukey’s HSD test)

### Relative weights and lengths of the intestine

On d 14, HW, SB, or their interaction had no significant effect on the relative weights of the intestine (*P* > 0.05; Table 3). However, there was a significant interaction between HW and SB for relative intestinal lengths (*P* < 0.05). The duodenum was shortest in the LHW-SB1 group and longest in the HHW-SB5 group (*P* = 0.001). The LHW-SB3 group had the shortest jejunum and ileum lengths, while the HHW-SB5 group had the longest lengths (*P* = 0.011 and *P* = 0.015, respectively). The relative cecal lengths were shorter in the LHW-SB1 and LHW-SB3 groups than in the HHW-SB5 group (*P* = 0.030). On d 42, HW, SB, and their interaction significantly affected various intestinal parameters (*P* < 0.05; Table 4). The jejunum relative weight was higher in the LHW category compared to the HHW category (*P* < 0.001), with the LHW-SB3 group showing the highest weight. Similar trends were observed for ileum and cecum weights, with higher values in the LHW category than in the HHW category. The ileum relative weight in the LHW category was higher in the LHW-SB1 group compared to the LHW-SB5 group (*P* = 0.013), while the cecum relative weight did not significantly differ among the LHW groups (*P* < 0.001). For the relative length of the jejunum, the LHW-SB3 group had the shortest length, and the HHW-SB5 group had the longest length (*P* < 0.001).

**Table 3:** Effects of in ovo sodium butyrate administration on the relative weights (g/100 g of body weight) and lengths (cm/100 g per body weight) of intestines in broiler chickens with different hatch weights on d 14

Items^1^		Relative weight				Relative length			
		Duodenum	Jejunum	Ileum	Cecum	Duodenum	Jejunum	Ileum	Cecum
HW	SB								
HHW	Control	1.71	1.92	1.25	0.84	5.64^cde^	11.99^b^	9.93^b^	5.38^abc^
	SB1	1.87	2.11	1.50	1.14	5.52^de^	12.05^b^	11.23^ab^	4.87^bc^
	SB3	1.72	2.18	1.43	1.05	6.38^ab^	12.64^b^	11.48^ab^	5.31^abc^
	SB5	1.84	2.67	1.74	0.85	6.94^a^	14.68^a^	13.31^a^	5.95^a^
LHW	Control	1.99	2.16	1.72	1.11	6.12^bcd^	12.89^ab^	11.74^ab^	5.66^ab^
	SB1	1.86	2.52	1.59	0.91	5.33^e^	11.82^b^	10.14^b^	4.59^c^
	SB3	1.68	2.20	1.52	1.08	5.73^bcde^	10.93^b^	9.85^b^	4.67^c^
	SB5	1.59	2.51	1.59	0.62	6.35^abc^	12.77^ab^	12.81^a^	5.01^abc^
SD		0.278	0.473	0.295	0.391	0.626	1.424	1.748	0.659
Main effects									
HW									
HHW		1.79	2.22	1.48	0.97	6.12	12.84^a^	11.49	5.38^a^
LHW		1.78	2.35	1.61	0.93	5.89	12.10^b^	11.14	4.98^b^
SB									
Control		1.85	2.04	1.49	0.98	5.88^b^	12.44^b^	10.84^b^	5.52^ab^
SB1		1.87	2.32	1.55	1.03	5.43^c^	11.94^b^	10.67^b^	4.73^c^
SB3		1.70	2.19	1.48	1.07	6.06^b^	11.79^b^	10.66^b^	4.99^bc^
SB5		1.72	2.59	1.67	0.74	6.65^a^	13.73^a^	13.06^a^	5.48^a^
*P* value									
HW		0.202	0.325	0.122	0.731	0.218	0.041	0.397	0.011
SB		0.165	0.323	0.328	0.160	< 0.001	< 0.001	< 0.001	0.001
HW × SB		0.202	0.187	0.062	0.332	0.001	0.011	0.015	0.030

^1^*HW *Hatch weight, *SB *Sodium butyrate inclusion level. Control: HHW or LHW chicks from eggs injected with 0.2 mL of 0.9% NaCl. SB1, SB3, SB5: HHW or LHW chicks from eggs injected with 0.2 mL of 0.1%, 0.3%, or 0.5% SB, respectively

The data are presented as the mean and pooled standard deviation (SD) (*n* = 6 birds/group)

^a–e^Values with different superscripts in a column indicate statistical significance at *P* < 0.05 (two-way ANOVA followed by Tukey’s HSD test)

**Table 4:** Effect of in ovo sodium butyrate administration on the relative weights (g/100 g of body weight) and lengths (cm/100 g per body weight) of intestines in broiler chickens with different hatch weights on d 42

Item^1^		Relative weight				Relative length			
		Duodenum	Jejunum	Ileum	Cecum	Duodenum	Jejunum	Ileum	Cecum
HW	SB								
HHW	Control	0.74	1.26^d^	0.92^d^	0.54^b^	1.45	3.19^bc^	3.20	1.69
	SB1	0.72	1.23^d^	0.90^d^	0.51^b^	1.44	3.27^abc^	3.42	1.62
	SB3	0.89	1.40^cd^	1.01^cd^	0.48^b^	1.43	3.23^bc^	3.52	1.68
	SB5	0.73	1.31^cd^	1.13^cd^	0.51^b^	1.41	3.62^ab^	3.46	1.67
LHW	Control	1.8	3.19^a^	2.13^ab^	1.17^a^	1.51	3.86^a^	3.58	1.84
	SB1	1.65	2.84^ab^	2.27^a^	1.08^a^	1.43	3.30^abc^	3.32	1.54
	SB3	1.44	3.26^a^	1.88^ab^	0.98^a^	1.26	2.93^c^	3.29	1.41
	SB5	1.47	2.04^bc^	1.61^bc^	0.83^a^	1.35	3.15^bc^	3.35	1.66
SD		0.487	0.896	0.619	0.301	0.132	0.419	0.485	0.246
Main effects									
HW									
HHW		0.77^b^	1.30^b^	0.99^b^	0.51^b^	1.43	333	3.40	1.67
LHW		1.59^a^	2.83^a^	1.97^a^	1.02^a^	1.39	3.31	3.39	1.61
SB									
Control		1.27	2.23^ab^	1.53	0.85^a^	1.48^a^	3.53^a^	3.39	1.77
SB1		1.18	2.04^b^	1.59	0.78^a^	1.43^b^	3.29^b^	3.37	1.58
SB3		1.16	2.33^a^	1.45	0.73^a^	1.35^c^	3.08^c^	3.41	1.55
SB5		1.10	1.68^c^	1.37	0.57^b^	1.38^b^	3.38^b^	3.42	1.67
*P* value									
HW		< 0.001	< 0.001	< 0.001	< 0.001	0.168	0.867	0.814	0.433
SB		0.399	0.028	0.456	< 0.001	0.046	0.021	0.247	0.154
HW × SB		0.076	< 0.001	0.013	< 0.001	0.162	< 0.001	0.389	0.137

^1^*HW* Hatch weight, *SB *Sodium butyrate inclusion level. Control: HHW or LHW chicks from eggs injected with 0.2 mL of 0.9% NaCl. SB1, SB3, SB5: HHW or LHW chicks from eggs injected with 0.2 mL of 0.1%, 0.3%, or 0.5% SB, respectively

The data are presented as the mean and pooled standard deviation (SD) (*n* = 6 birds/group)

^a–d^Values with different superscripts in a column indicate statistical significance at *P* < 0.05 (two-way ANOVA followed by Tukey’s HSD test)

### Gene expression in intestinal mucosa

#### Jejunum

**Fig. 2:**
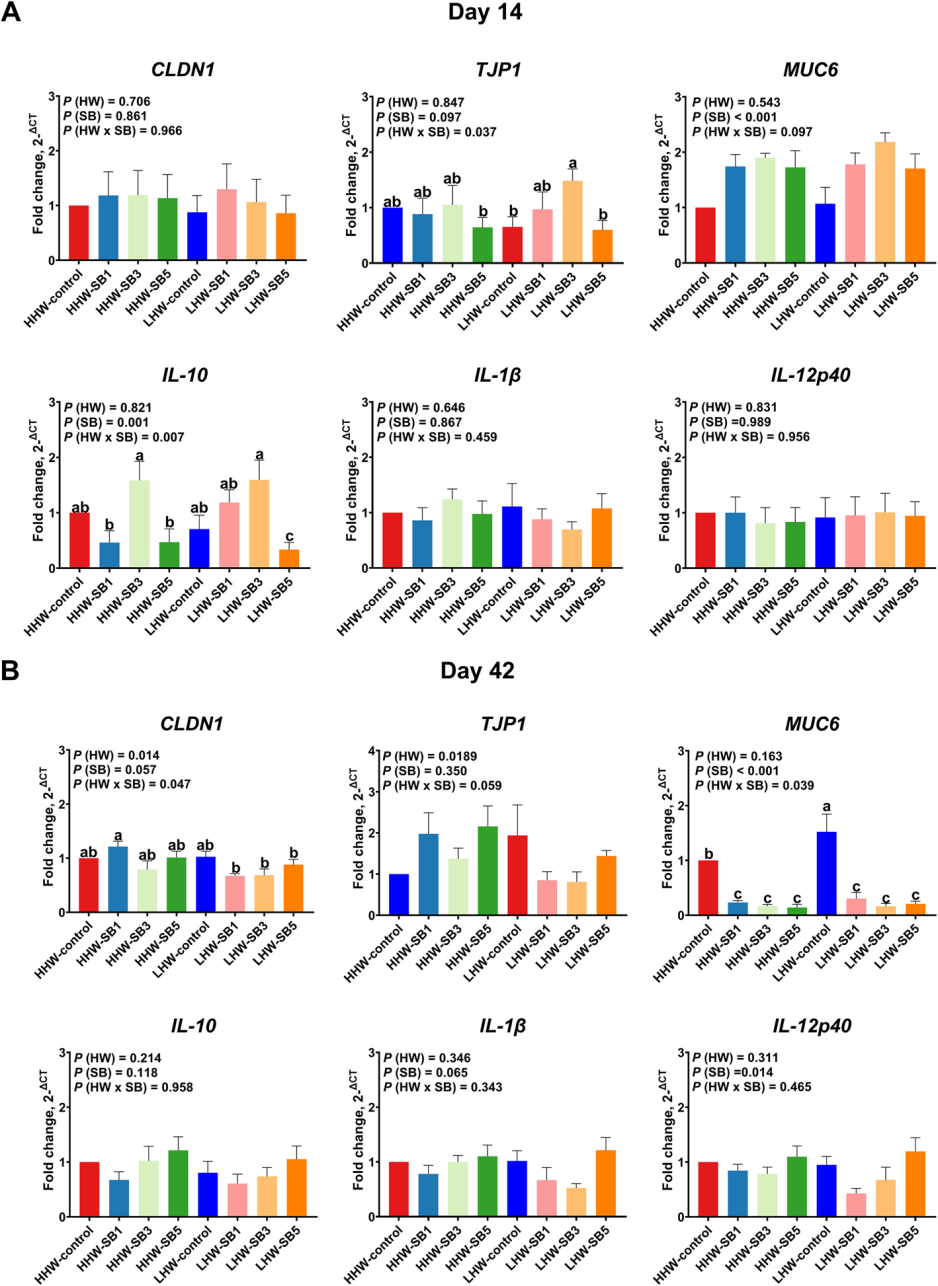
The relative expression of gut barrier and immune-related genes in the jejunal mucosa of high (HHW) and low (LHW) hatch weight chickens on d 14 (**A**) and d 42 (**B**) that had received 3 levels of sodium butyrate in ovo (SB1: 0.1%, SB3: 0.3%, SB5: 0.5%) or 0.9% NaCl (control) in ovo. Two-way ANOVA was applied to determine the fold change of the relative expression of genes (*n* = 6 birds/group) and *P* values are indicated by different letters corresponding to *P *(HW), *P* (SB), and *P* (HW × SB). ^a^^–^^c^Values with different letters indicate statistical significance at *P* < 0.05

On d 14, the expression of *TJP1* and *IL-10* in the jejunum was significantly influenced by the interaction between HW and SB (*P* = 0.037 and *P* = 0.007, respectively; Fig. 2A), with the LHW-SB3 group exhibiting the highest expression and the LHW-SB5 group exhibiting the lowest expression. *MUC6* expression was affected by SB (*P* < 0.001), with SB3-treated groups showing higher levels regardless of HW. On d 42, significant interactions between HW and SB were observed for *CLDN1* and *MUC6* expressions (*P* = 0.047 and *P* = 0.039, respectively; Fig. 2B). The HHW-SB1 group had higher *CLDN1* expression compared to all the LHW groups receiving in ovo SB injection but did not differ from the LHW and HHW control groups. *MUC6* expression was lower in birds receiving in ovo SB injections (both LHW and HHW) than in control birds, with the LHW-control group showing the highest *MUC6* expression. *IL-12p40* expression revealed a significant main effect of SB (*P* = 0.014), with the SB5-treated groups exhibiting greater expression than the other groups.

#### Ileum

**Fig. 3:**
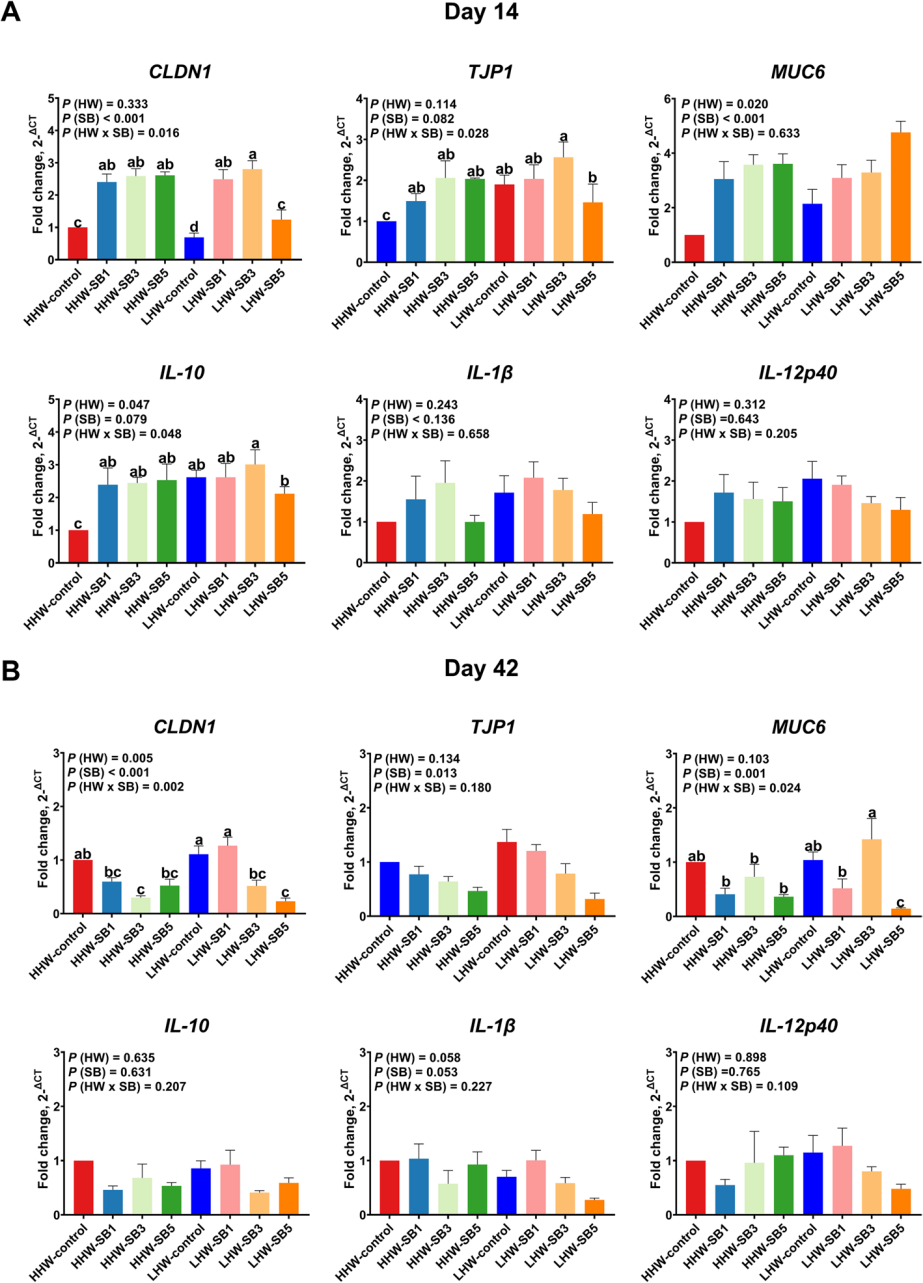
The relative expression of gut barrier and immune-related genes in the ileal mucosa of high (HHW) and low (LHW) hatch weight chickens on d 14 (**A**) and d 42 (**B**) that had received 3 levels of sodium butyrate in ovo (SB1: 0.1%, SB3: 0.3%, SB5: 0.5%) or 0.9% NaCl (control) in ovo. Two-way ANOVA was applied to determine the fold change of the relative expression of genes (*n* = 6 birds/group) and *P* values are indicated by different letters corresponding to *P *(HW), *P *(SB), and *P *(HW×SB). ^a^^–^^d^Values with different letters indicate statistical significance at *P* < 0.05

On d 14, the expression of *CLDN1*, *TJP1*, and *IL-10* in the ileum was significantly influenced by the interaction between HW and SB treatment (*P* = 0.016, *P* = 0.028, and *P* = 0.048, respectively; Fig. 3A). The LHW-SB3 group showed the highest *CLDN1* expression compared to the LHW and HHW control groups, and the LHW-control group had lower *CLDN1* levels than the HHW-control group. *TJP1* expression was higher in the LHW-SB3 group than in the HHW-control and LHW-SB5 groups, though not significantly different from other groups. All in ovo SB groups had higher *IL-10* expression compared to the HHW-control group, with the LHW-SB3 group showing the highest levels. The LHW-control group also had higher *IL-10* expression than the HHW-control group. For *MUC6* expression, LHW chicks that received in ovo SB had higher levels, which increased with increasing SB dose (*P* < 0.05). On d 42, significant interactions between HW and SB were seen for *CLDN1* and *MUC6* (*P* = 0.002 and *P* = 0.024, respectively; Fig. 3B). The LHW-SB1 group had higher *CLDN1* levels than all other in ovo SB groups, with no significant difference from the LHW and HHW controls. The LHW-SB3 group had the highest *MUC6* expression among all the SB groups.

#### Cecum

**Fig. 4:**
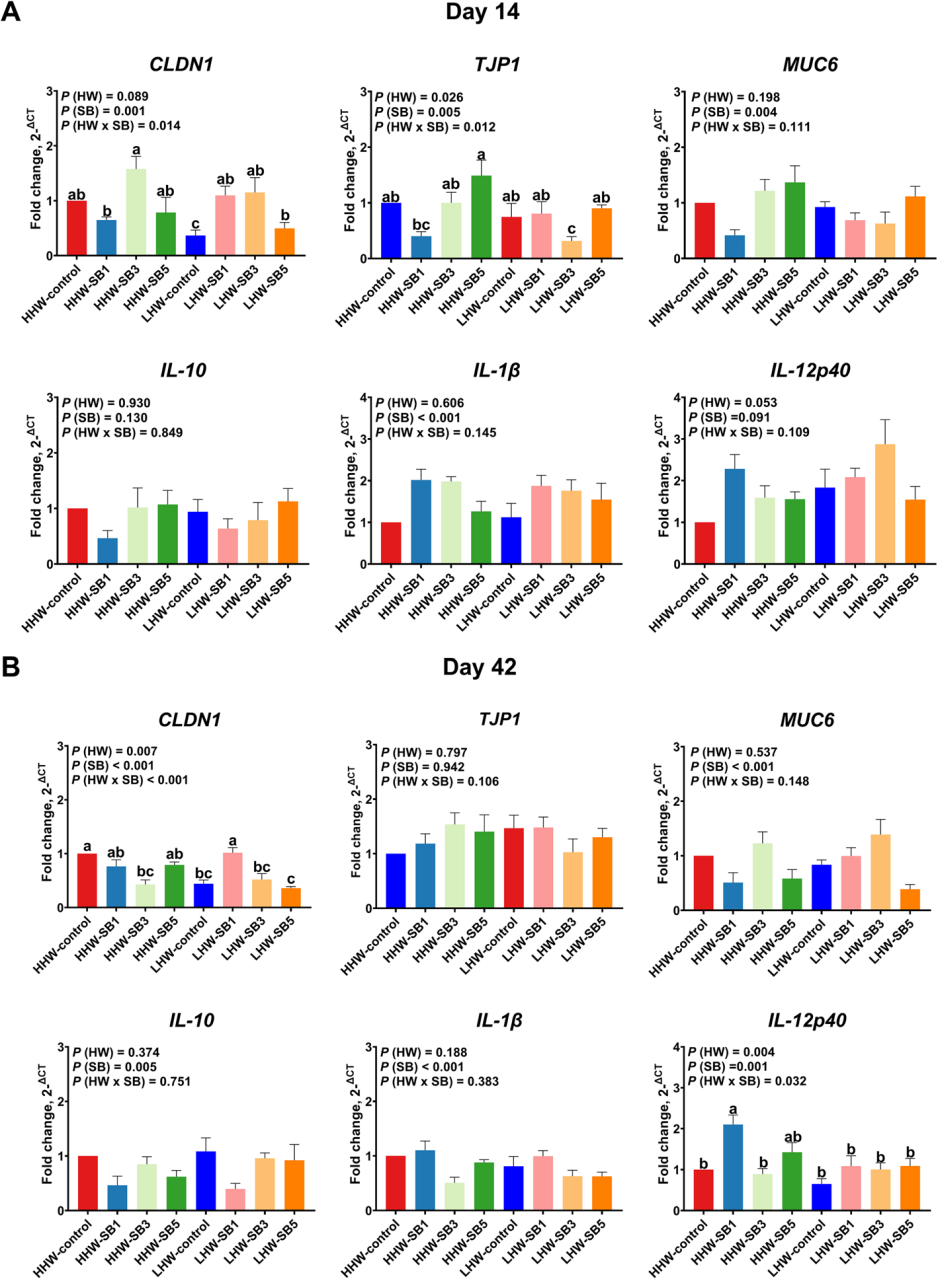
The relative expression of gut barrier and immune-related genes in the cecal mucosa of high (HHW) and low (LHW) hatch weight chickens on d 14 (**A**) and d 42 (**B**) that had received 3 levels of sodium butyrate in ovo (SB1: 0.1%, SB3: 0.3%, SB5: 0.5%) or 0.9% NaCl (control) in ovo. Two-way ANOVA was applied to determine the fold change of relative expression (*n* = 6 birds/group) and *P* values are indicated by different letters corresponding to *P* (HW), *P* (SB), and *P* (HW×SB). ^a^^–^^c^Values with different letters indicate statistical significance at *P* < 0.05

On d 14, *CLDN1* and *TJP1* expressions were affected by HW and SB interactions (*P* = 0.014 and *P* = 0.012, respectively; Fig. 4A), with HHW-SB3 showing higher *CLDN1* expression than all the other groups. *TJP1* expression was significantly upregulated in the HHW-SB5 group compared to the HHW-SB1 and LHW-SB3 groups. SB treatment also had a significant main effect on *MUC6* and *IL-1β* expressions (*P* = 0.004 and *P* < 0.001). *MUC6* expression increased with increasing SB dose, while *IL-1β* decreased with increasing dose. On d 42, *CLDN1* and *IL-12p40* expressions were significantly influenced by the interaction between HW and SB (*P* < 0.001 and *P* = 0.032, respectively; Fig. 4B). The LHW-SB1 group exhibited the highest *CLDN1* expression, which did not differ significantly from that of the HHW-SB1 and HHW-control groups. Additionally, the LHW-control group presented lower *CLDN1* expression than the HHW-control group. The HHW-SB1 group showed higher expression of *IL-12p40* than all the other groups, except for the HHW-SB5 group. SB treatment also had a significant main effect on *MUC6* expression (*P* < 0.001), with SB3-treated groups showing higher levels regardless of HW.

### Microbiota analysis

#### Temporal changes and core microbiota composition

**Fig. 5:**
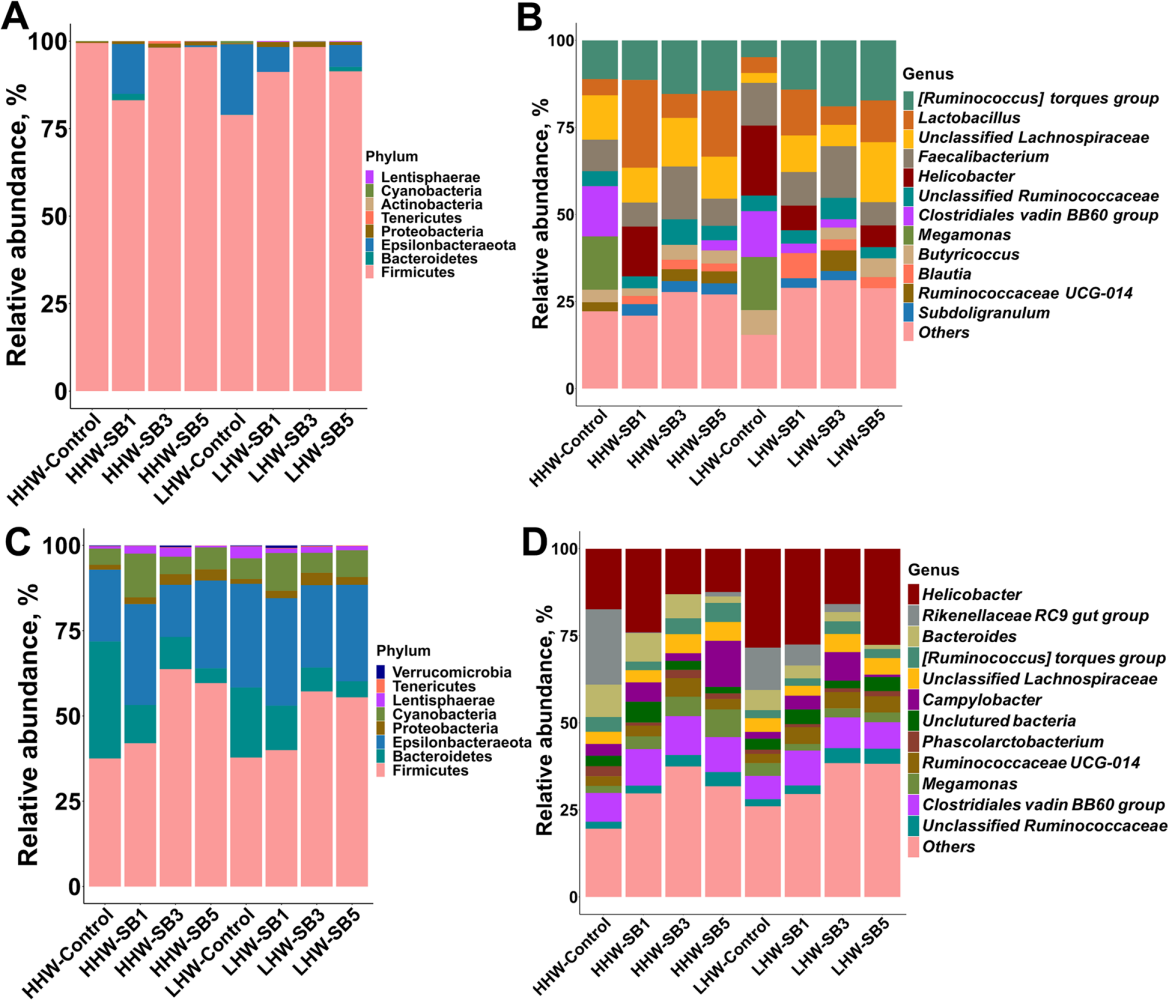
Relative abundance of cecal bacterial phyla and genera in high (HHW) and low (LHW) hatch weight chickens on d 14 (**A** and **B**) and 42 (**C** and **D**) that had received 3 levels of sodium butyrate (SB1: 0.1%, SB3: 0.3%, SB5: 0.5%) or 0.9% NaCl (control) in ovo. The data are from individually sampled chickens (*n* = 6 birds/group)

Cecal metagenome sequencing generated 3,971,213 reads, with 41,366 ± 28,810 (mean ± SD) reads per sample. After quality filtering, 2,573,048 reads remained, with an average of 26,802 reads per sample. Compositional analysis revealed considerable inter-individual variability and a significant shift in gut microbiota from d 14 to d 42 post-hatch (Fig. 5). On d 14, the microbiota was dominated by Firmicutes phylum (79%–99%), with minor contributions from Epsilonbacteraeota (0–20%), Proteobacteria (0.2%–1.5%), and Bacteroidota (0–1.8%, Fig. 5A). As the chickens matured to d 42, Firmicutes remained the most abundant phylum but its dominance decreased substantially (38%–64%), leading to a significant increase in Bacteroidetes (4%–34%) and Epsilonbacteraeota (15%–32%, Fig. 5C). A few low-abundance previously undetected phyla also emerged at this stage, including Cyanobacteria (5%–13%), Lentisphaerae (0.5%–3.5%), Tenericutes (0.1%–0.2%) and Verrucomicrobia (0.004%–0.76%), indicating diversification of the microbial ecosystem. At the genus level, on d 14, prominent early colonizers such as *Lactobacillus* (5%–25%), unclassified *[Ruminococcus] torque group* (4.8%–19%), unclassified *Lachnospiraceae* (3%–17%) and *Faecalibacterium* (7%–15%) were observed (Fig. 5B). By d 42, the *Lactobacillus*-dominated community had transitioned to one where *Helicobacter* was the most prevalent genus (12%–28%), and this change was accompanied by an increase in the *Rikenellaceae RC9 gut group* (0–22%), *Campylobacter* (0.7%–13%), and *Clostridiales vadinBB60 group* (5.4%–9.5%, Fig. 5D). Despite the consistency of core genera across individuals, many low-abundance genera collectively made up more than 20% of the community on both days, representing a highly variable component of the gut ecosystem.

#### Alpha and beta diversity

**Fig. 6:**
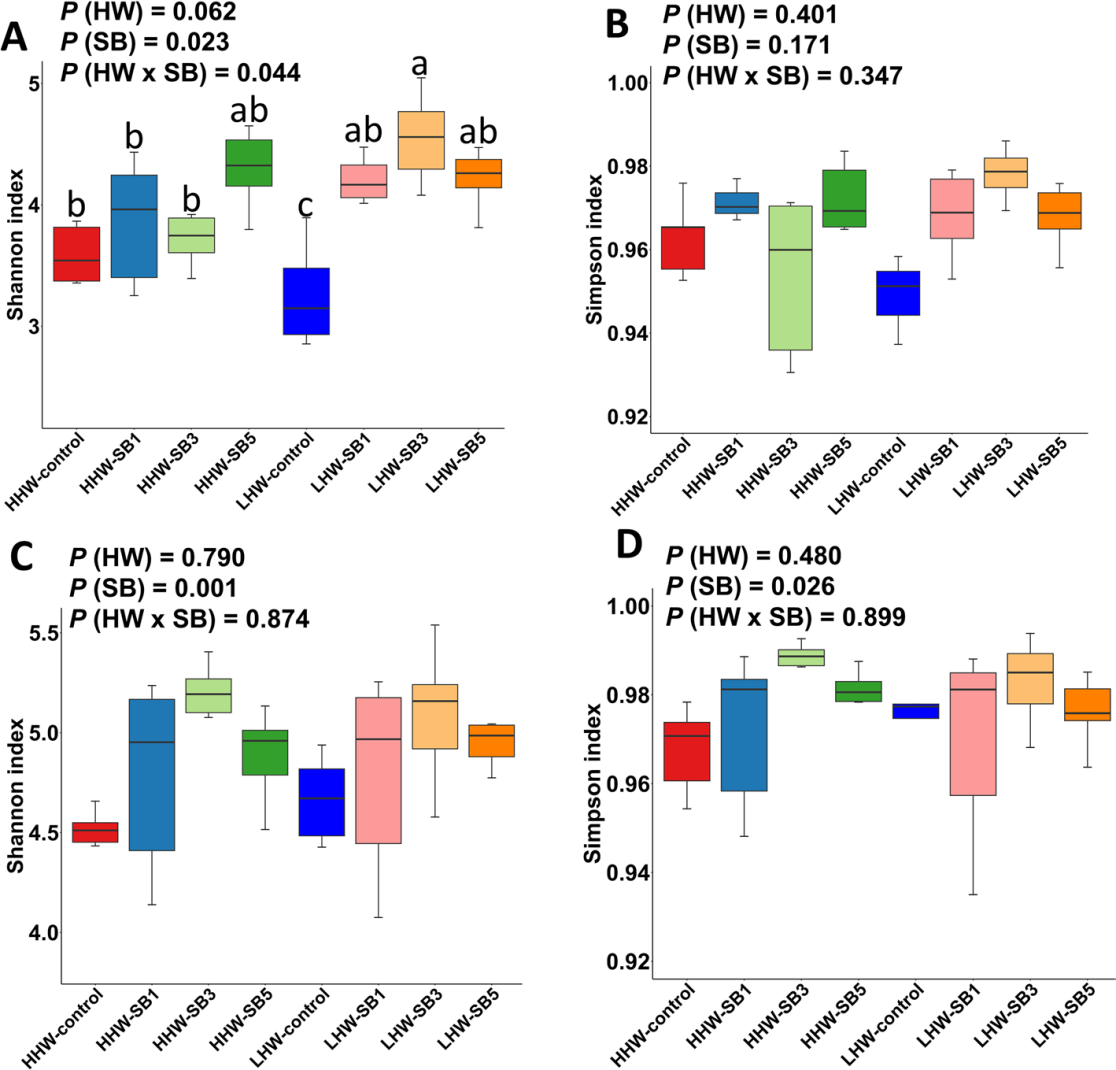
Alpha diversity of the cecal microbiota, measured using the Shannon and Simpson indexes, in high (HHW) and low (LHW) hatch weight chickens on d 14 (**A** and **B**) and d 42 (**C** and **D**) that had received 3 levels of sodium butyrate in ovo (SB1: 0.1%, SB3: 0.3%, SB5: 0.5%) or 0.9% NaCl (control) in ovo. Two-way ANOVA was applied to the alpha diversity metrics (*n* = 6 birds/group) and *P* values are indicated by different letters corresponding to *P* (HW), *P* (SB), and *P* (HW×SB). ^a^^–^^c^Values with different letters indicate statistical significance at *P* < 0.05

**Fig. 7:**
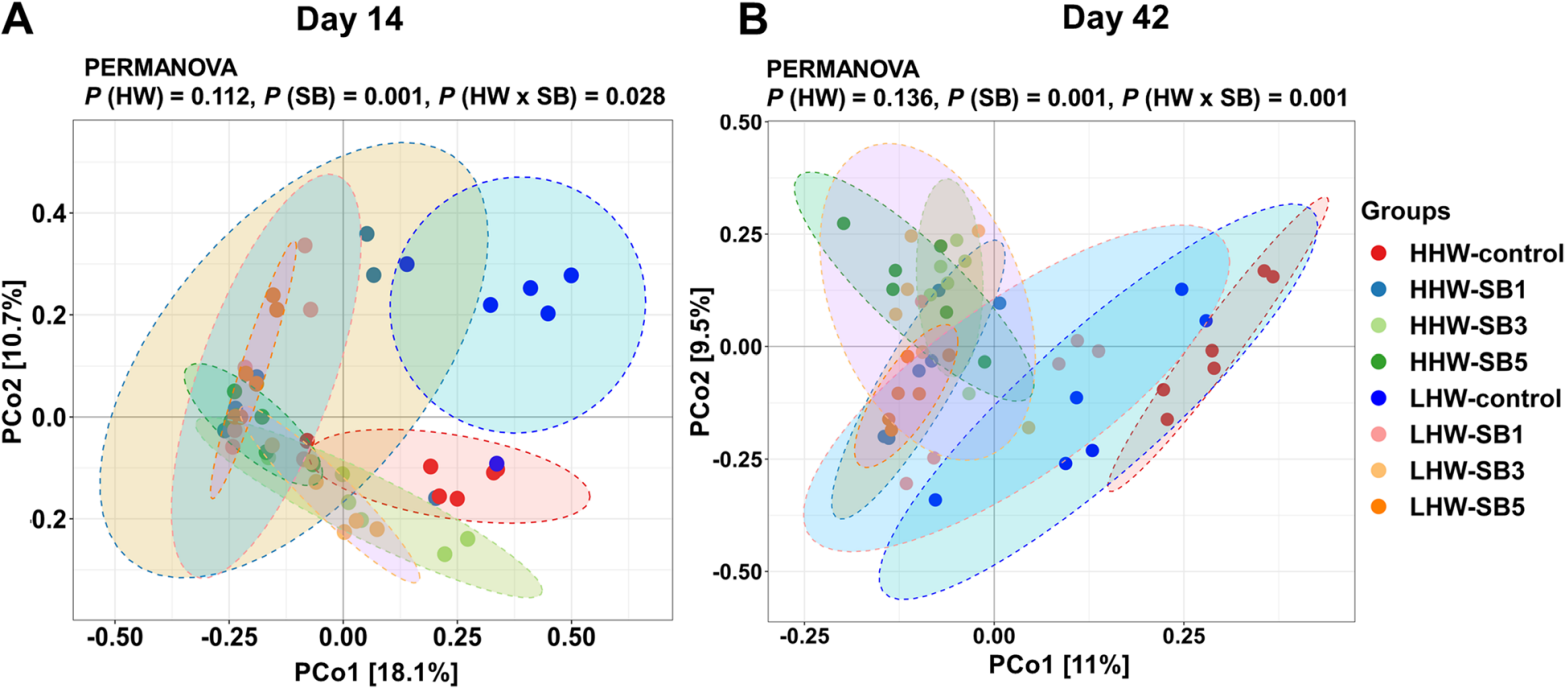
Principal coordinate analysis (PCoA) generated based on Bray‒Curtis distance comparing the gut microbiota composition of high (HHW) and low (LHW) hatch weight chickens on d 14 (**A**) and d 42 (**B**) that had received 3 levels of sodium butyrate (SB1: 0.1%, SB3: 0.3%, SB5: 0.5%) or 0.9% NaCl (control) in ovo. A nonparametric permutational multivariate analysis of variance (PERMANOVA) was applied to the Bray–Curtis distance and *P* values are indicated by different letters corresponding to *P* (HW), *P* (SB), and *P* (HW×SB)

On d 14, the Shannon index of alpha diversity showed a significant interaction between HW and SB (*P* = 0.044; Fig. 6A). The Shannon index was highest in the LHW-SB3 group, while it was lowest in the LHW-control group. The HHW-control group also had a greater Shannon index than the LHW-control group. On d 42, SB had a significant effect on both the Shannon and Simpson indexes (*P* < 0.05; Fig. 6C and D), with SB3-treated groups showing higher values regardless of HW. However, the HW and HW × SB interactions did not significantly affect alpha diversity on d 42. Beta diversity analysis via PERMANOVA of the Bray‒Curtis distance showed significant HW × SB interaction effects on the microbiota composition on both d 14 (*P* = 0.028) and d 42 (*P* < 0.001; Fig. 7A and B). The control groups (LHW and HHW) formed distinct clusters, while the SB-treated groups exhibited similar clusters, indicating that SB had a homogenizing effect on the microbiota composition. The Bray–Curtis dissimilarity boxplot also showed that the SB-treated groups had microbiota profiles closer to each other than the LHW and HHW control groups (Fig. S2).

#### Differential abundance of bacterial genera

**Fig. 8:**
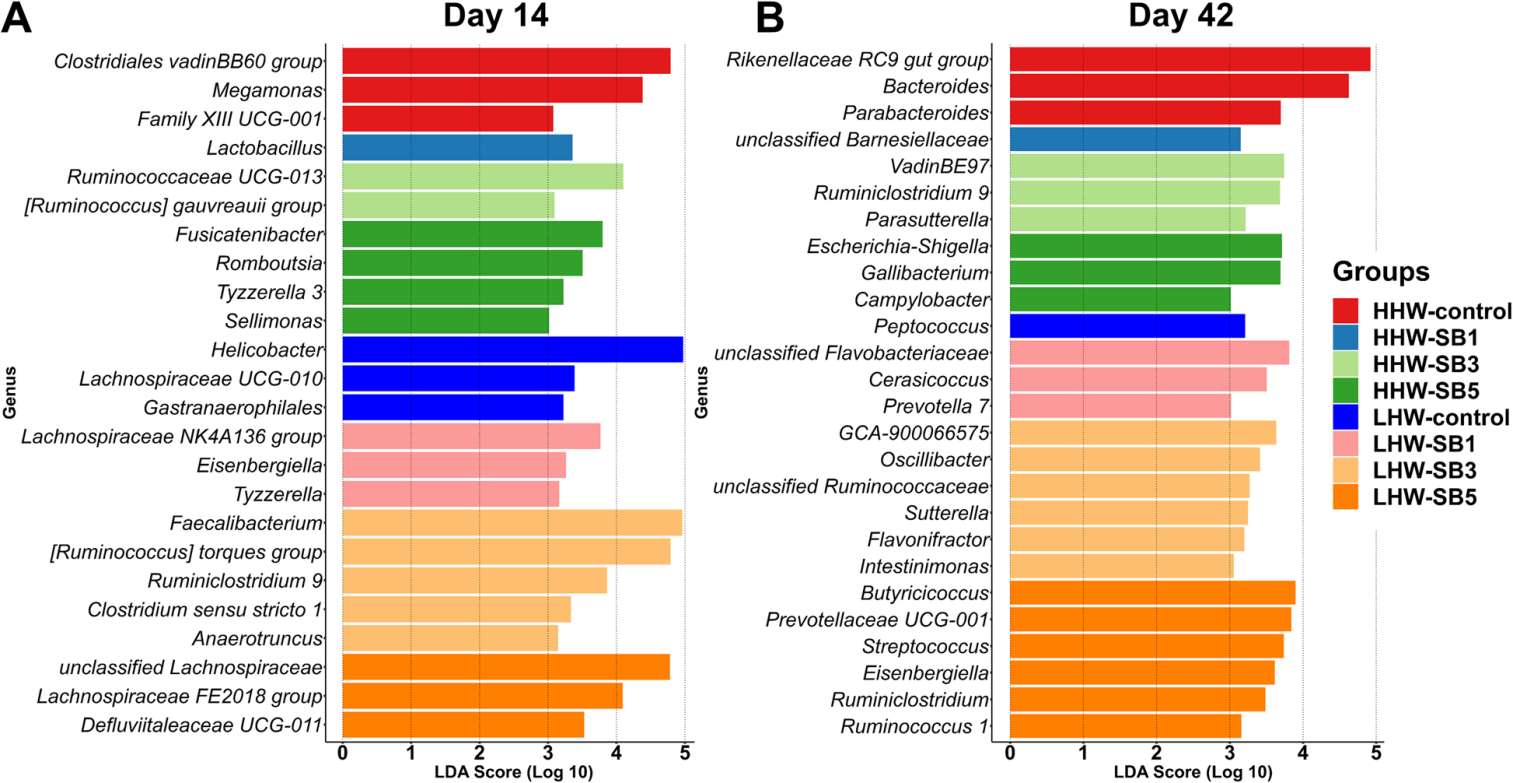
Differentially enriched cecal bacterial genera in high (HHW) and low (LHW) hatch weight chickens on d 14 (**A**) and d 42 (**B**) that had received 3 levels of sodium butyrate (SB1: 0.1%, SB3: 0.3%, SB5: 0.5%) or 0.9% NaCl (control) in ovo. LEfSe analysis was performed (*n* = 6 birds/group) using an FDR < 0.05 and a linear discriminate analysis (LDA) score of ≥ 3.0 as the thresholds

On d 14, LEfSe analysis identified 24 differentially abundant genera across all groups (Fig. 8A, LDA cut-off value ≥ 3.0, FDR < 0.05). In the HHW category, the control group exhibited enrichment of the *Clostridial vadinBB60 group*, *Megamonas*, and *Family XIII UCG-001*. The HHW-SB1 group showed higher *Lactobacillus* abundance. The HHW-SB3 group was enriched in the *Ruminococcaceae UCG-013* and *[Ruminococcus] gauvreauii group*. The HHW-SB5 group presented high enrichment of *Fusicatenibacter*, *Romboutsia*, *Tyzzerella 3*, and *Sellimonas*. In the LHW category, the control group exhibited differential abundances of *Helicobacter*, *Lachnospiraceae UCG-010*, and *Gastranaerophilales*. The abundance of *Lachnospiraceae NK4A136 group*, *Eisenbergiella*, and *Tyzzerella* was increased in the LHW-SB1 group. The LHW-SB3 group was enriched in *Faecalibacterium*, *[Ruminococcus] torques group*, *Ruminiclostridium 9*, and *Anaerotruncus*. The LHW-SB5 group had higher abundance of *unclassified Lachnospiraceae*, *Lachnospiraceae FE2018*, and *Defluviitaleaceae UCG-011*.

On d 42, the analysis revealed 26 bacterial genera exhibiting differential abundance among all groups (Fig. 8B, LDA cut-off value ≥ 3.0, FDR < 0.05). In the HHW category, the control group demonstrated enrichment of the *Rikenellaceae RC9 gut group*, *Bacteroides*, and *Parabacteroides*. The HHW-SB1 group showed higher *unclassified Barnesiellaceae*, while HHW-SB3 had increased VadinBE97, *Ruminiclostridium 9*, and *Parasutterella* abundances. The HHW-SB5 group had a greater abundance of *Escherichia-Shigella*, *Gallibacterium*, and *Campylobacter*. In the LHW category, the control group showed enrichment of *Peptococcus*. The LHW-SB1 group exhibited higher abundance of unclassified *Flavobacteriaceae*, *Cerasicoccus*, and *Prevotella 7*. The LHW-SB3 group had a higher abundance of *GCA-900066575*, *Oscillibacter*, *unclassified Ruminococcaceae*, *Sutterella*, *Flavonifractor*, and *Intestinimonas*. The LHW-SB5 group exhibited increased abundances of *Streptococcus*, *Eisenbergiella*, *Ruminiclostridium*, and *Ruminococcus 1*.

#### Predicted functionality of the cecal microbiota

Metabolic pathway analysis using the MetaCyc database identified 321 pathways on d 14 and 315 pathways on d 42 across all groups. Two-way hierarchical clustering of the top 50 pathways, including those related to fermentation, sugar metabolism, amino acid biosynthesis, genetic processing, and cell wall components, revealed distinct groupings (Fig. S3). On d 14, clustering revealed 3 groups: LHW-SB3 and HHW-SB3 clustered together with higher levels of amino acid biosynthesis and galactose and starch degradation pathways; HHW-control and LHW-control formed another cluster; and the remaining groups were separated (Fig. S3A). By d 42, HHW-SB3 exhibited a distinct pattern of decreased amino acid biosynthesis, while LHW-SB1 and HHW-SB1 grouped together with higher activity in genetic processing and cell wall pathways (Fig. S3B).

**Fig. 9:**
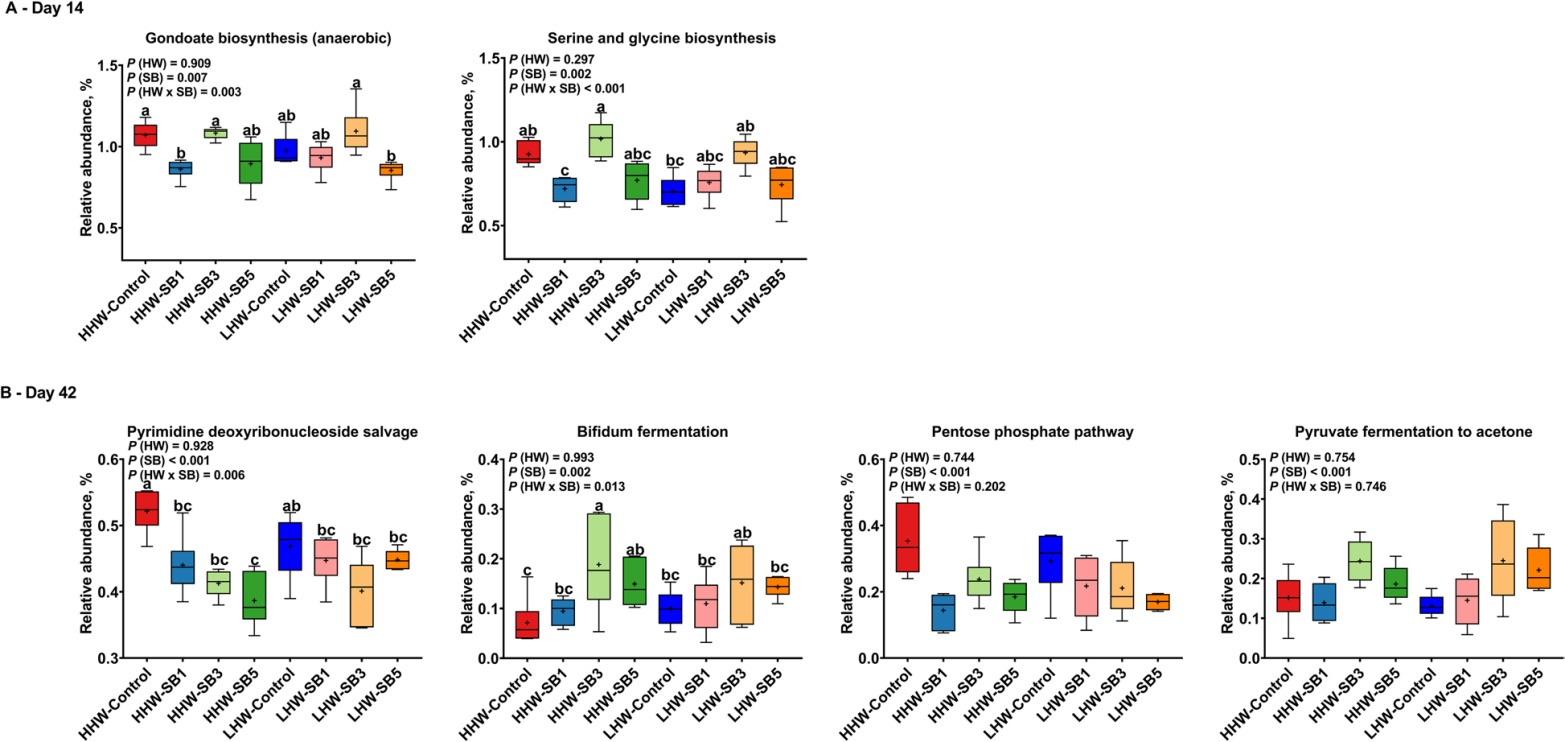
Predicted microbial metabolic pathways of high (HHW) and low (LHW) hatch weight chickens on d 14 (**A**) and d 42 (**B**) that had received 3 levels of sodium butyrate (SB1: 0.1%, SB3: 0.3%, SB5: 0.5%) or 0.9% NaCl (control) in ovo. Only significantly different metabolic pathways are shown (*P* value < 0.05)

 Two-way ANOVA revealed significant differences in only two metabolic pathways on d 14 and 4 pathways on d 42 (Fig. 9). On d 14, the LHW-SB3 group exhibited a greater abundance of the gondoate biosynthesis pathway, though not significantly different from HHW-SB3 group (Fig. 9A). The HHW-SB3 group had the highest levels of microbial genes involved in serine and glycine biosynthesis, while the HHW-SB1 group had the lowest. On d 42, the HHW-control group had a greater abundance of the pyrimidine deoxyribonucleoside salvage pathway, and HHW-SB3 showed greater enrichment of the bifidum fermentation pathway (Fig. 9B).

#### Correlations between bacterial genera and metabolic pathways

**Fig. 10:**
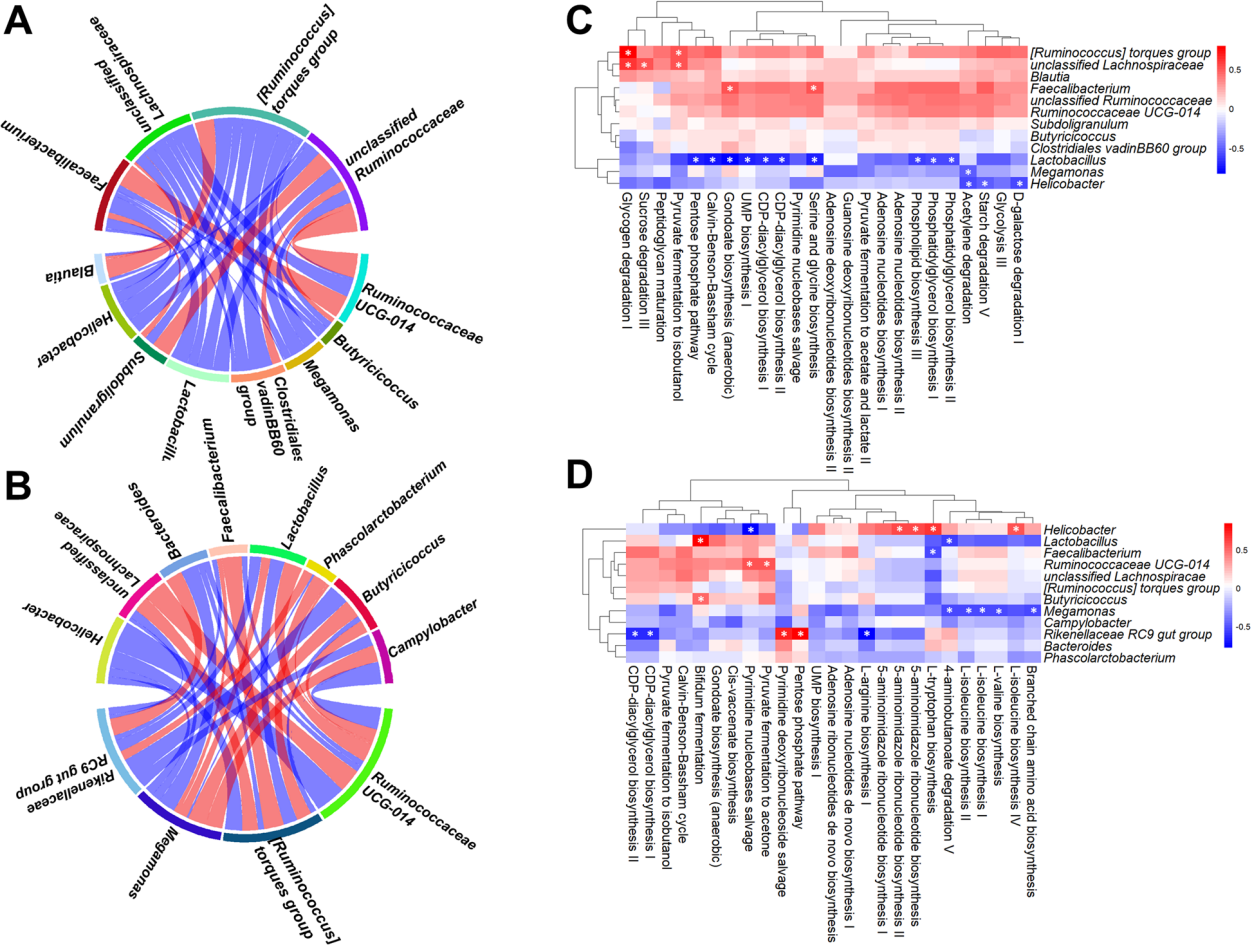
Chord diagram showing Pearson’s correlations between the most abundant bacterial genera on d 14 (**A**) and d 42 (**B**). Chord width reflects the strength of the correlations, with red indicating positive correlations and blue indicating negative correlations. Heatmap of Pearson’s correlations between specific bacteria and the 25 most abundant predicted metabolic pathways on d 14 (**C**) and d 42 (**D**). Significant correlations (*P* < 0.05) are marked with an asterisk, with red representing positive correlations and blue representing negative correlations

 Pearson’s correlation analysis revealed relationships among the top 12 most abundant bacterial genera and 25 metabolic pathways (Fig. 10). On d 14, *unclassified Ruminococcaceae* were positively correlated with several genera, including *Subdoligranulum* and *Ruminococcaceae UCG-014* (Fig. 10A). *Ruminococcaceae UCG-014* was positively correlated with *Faecalibacterium*, while Blautia was positively correlated with the *[Ruminococcus] torques group*. *Helicobacter* and *Lactobacillus* were negatively correlated with most genera. On d 42, the *[Ruminococcus] torques group* was positively correlated with *Faecalibacterium*, and *unclassified Lachnospiraceae* was positively correlated with *Ruminococcaceae UCG-014* (Fig. 10B). Most genera maintained negative correlations with *Helicobacter*. For metabolic pathways, on d 14, *Lactobacillus*, *Megamonas*, and *Helicobacter* formed a distinct cluster with negative correlations with most pathways (Fig. 10C). *Faecalibacterium* positively correlated with gondoate biosynthesis and serine-glycine biosynthesis, while *unclassified Lachnospiraceae* and the *[Ruminococcus] torques group* positively correlated with pyruvate fermentation to isobutanol and glycogen degradation I. On d 42, *Megamonas* was negatively correlated with most pathways. *Helicobacter* was positively correlated with L-isoleucine and L-tryptophan biosynthesis but negatively correlated with pyrimidine nucleobase salvage (Fig. 10D). *Lactobacillus* abundance was negatively correlated with 4-aminobutanoate degradation V but was positively correlated with bifidum fermentation. The *Rikenellaceae RC9 gut group* was positively correlated with several pathways, including those related to the pentose phosphate pathway and pyrimidine deoxyribonucleoside salvage and was negatively correlated with L-arginine biosynthesis I.

## Discussion

The current study revealed a significant impact of HW on broiler performance, showing substantial advantages for HHW chicks over their LHW counterparts when both were administered physiological saline as a control. HHW-control group exhibited higher BW, ADG, and improved FCR compared to LHW-control group, consistent with previous studies reporting that HHW chicks generally outperform LHW chicks on control diets [5, 27].

The hatchability did not differ across treatments, which suggested that none of the treatments adversely affected embryonic viability. In the present study, in ovo SB administration demonstrated growth-promoting effects by improving ADG and the FCR, which is consistent with the findings of previous reports on the positive impacts of dietary supplementation or in ovo butyrate administration on broiler performance [11, 28]. However, the effects were dose dependent. The 0.3% SB dose produced the most favorable results, which was consistent with the findings of Saleha et al. [29], who reported that weight gain and performance improved with this dose. In contrast, a higher dose of 0.5% proved too high for in ovo stimulation, negatively affecting ADG and FCR, suggesting an optimal dose range for SB beyond which negative impacts may occur. Pineda-Quiroga et al. [30] reported similar observations, finding that high inclusion rates of SB decreased broiler weight and feed intake while worsening the FCR at every growth stage.

In ovo SB administration influenced growth in both HW categories. However, the beneficial effects were more pronounced in LHW broilers at the 0.3% inclusion level, suggesting that the optimal SB dosage might help bridge the performance gap between LHW and HHW chicks. SB may improve weight gain in chickens by upregulating nutrient transporter activity, stimulating intestinal cell proliferation, modulating tight junction protein expression, and improving nutrient digestibility [31–33]. It also creates an acidic environment in the gut, which minimizes the load of pathogens [10]. During the early post-hatching period, butyrate production in the intestines is generally insufficient due to inadequate microbiota colonization [34]. This deficiency is likely more severe in low-weight chicks, which are known to have compromised gut health and an unbalanced microbiota composition compared to their heavier counterparts [3, 7, 8]. In ovo SB injection likely addressed this deficiency by supplying an optimal amount of butyrate at a critical developmental stage, thereby improving gut function and overall performance in LHW chicks. In contrast, SB has shown limited effects on production and gut health parameters in healthy and unchallenged chickens [35–37]. Therefore, HHW chicks would have been less responsive to SB marginal benefits, as high-performing broilers typically have better initial gut development and face fewer gut-related challenges [8].

The study also revealed divergent effects of SB on intestinal development between HW categories. The HHW-SB5 group, despite suboptimal performance, had longer intestines, contradicting the typical correlation between longer intestines and improved nutrient absorption [10]. In contrast, the LHW-SB3 group, despite having shorter intestines, exhibited better performance, suggesting a potential metabolic nutrient-saving mechanism induced by SB, where energy is redirected from intestinal maintenance to growth and muscle development. Furthermore, the LHW-SB3 group had a greater relative jejunum weight on d 42, likely due to the trophic effect of butyrate on epithelial cells, which enhances cell proliferation, differentiation, and maturation [38], resulting in an increase in absorptive surface area.

In ovo SB administration led to increased expression of *CLDN1*, *TJP1*, and *MUC6* across various intestinal segments, suggesting that SB may protect the mucosal epithelium from injury and alleviate enteropathic stress by enhancing gut barrier function and mucus secretion. Song et al. [31] similarly found that in feed butyrate administration has a protective effect in necrotic enteritis-challenged broilers by alleviating gut barrier injuries through the upregulation of the jejunal *CLDN1*, *CLDN4* and *occludin* genes. Butyrate enhances intestinal barrier function by accelerating the assembly of tight junctions through AMP-activated protein kinase activation [39], which suggests that SB induces epithelial cell differentiation toward tight junction cells, which could improve intestinal health and integrity. Although gut barrier-related gene expression also increased in other groups, the LHW-SB3 group exhibited the most pronounced upregulation, indicating a particularly beneficial effect on gut barrier function in these chickens. The divergent responses of the HHW and LHW categories to SB injection could be related to differences in intestinal health and development. Butyrate tends to exert more significant effects under stressful conditions [31, 40, 41] but has minimal impact on the gut epithelium of healthy chickens [35]. Since low-weight chickens often face gut health challenges such as delayed GIT development and compromised barrier function [8], SB injection likely benefits them more than their heavier counterparts. Our observations also revealed varying immune responses among different HW categories following in ovo injection of SB. IL-10 is a potent anti-inflammatory cytokine produced by activated macrophages that plays a crucial role in enhancing intestinal barrier function and attenuating intestinal inflammation [42]. LHW chicks receiving 0.3% SB showed increased *IL-10* expression, suggesting that localized anti-inflammatory effects likely contributed to enhanced gut health. Conversely, 0.5% SB in HHW chicks resulted in increased *IL-12p40* expression, indicating potential inflammation. IL-12p40, a subunit of IL-12, is involved in regulating cell-mediated immune responses and inducing inflammatory mediators [43]. It is well established that overwhelming production of pro-inflammatory cytokines is energetically expensive due to the metabolic demands on immune cells and the negative effects of prolonged inflammation such as anorexia and tissue degradation [37].

Cecal microbiota analysis revealed a shift from a Firmicutes-dominated community to a more diverse ecosystem with increased Bacteroidetes abundance over time, consistent with the findings of previous studies [44]. Our study revealed greater alpha diversity in the HHW-control group on d 14 than in the LHW-control group, suggesting an advantage in gut microbial development for heavier chicks [45]. Consistent with the findings of previous studies [46], SB injection significantly impacted the biodiversity of the microbiota in both HW categories, with the LHW-SB3 group showing the highest Shannon index of alpha diversity on d 14. This increased diversity, particularly in LHW chicks, may be crucial for improving gut health and performance, as higher bacterial diversity is linked to better gut health and infection resistance [2]. PCoA further showed that in ovo SB administration resulted in a significant separation of microflora, implying that SB altered the composition of the flora compared to the controls.

LEfSe analysis revealed that the HHW-control group exhibited a greater proportion of beneficial bacteria, including the genus *Megamonas*, which plays a crucial role in fermenting glucose into acetate and propionate, as well as cellulose-degrading bacteria such as the *Clostridiales vadin BB60 group*, *Bacteroides*, *Parabacteroides*, and the *Rikenellaceae RC9 gut group* [47–49]. In addition, *Bacteroides* has immune-modifying functions and inhibits inflammatory cytokines [50]. The LHW-control group had an increased abundance of the pathogenic *Helicobacter* genus, implying that *Helicobacter* species, particularly *Helicobacter pylori* and *Helicobacter pullorum*, are known to negatively impact GIT structure, health, and growth performance in broilers [7, 51]. These pathogens may cause gastroenteritis in chickens and pose potential risks to human health through meat contamination [52]. The LHW-SB3 group had a greater abundance of the genus *Faecalibacterium*, a genus associated with high performance in male broilers [3], and a reduced abundance of this genus is often linked to inflammatory diseases [53]. *Faecalibacterium prausnitzii*, the only known species in this genus, is a potent butyrate producer and probiotic in livestock [54]. A correlation analysis showed that *Faecalibacterium* was positively correlated with several predicted metabolic pathways, including gondoate biosynthesis, a known antimicrobial agent against Gram-negative pathogenic bacteria [55], which is beneficial to host health. Other taxa in this group that contribute to SCFA production and weight gain included *Flavonifractor*,* [Ruminococcus] torques group*, *Ruminococcaceae UCG-10*, *Anaerotruncus*, *Intestinimonas*, *Sutterella*, and *Oscillibacter* [56–58]. An increase in the proportion of these beneficial bacteria in the LHW-SB3 group was expected to positively impact intestinal health and overall performance. However, the 0.5% SB treatment in the HHW group resulted in higher abundances of pathogenic genera such as *Escherichia-Shigella*, *Galibacterium*, and *Campylobacter*, which might be correlated with their limited growth response and increased expression of pro-inflammatory cytokine *IL-12p40*. *Gallibacterium anatis*, a Gram-negative bacterium from the Pasteurellaceae family, typically resides in the respiratory and reproductive tracts, and significantly impacts animal welfare and productivity by causing peritonitis and mortality [59]. Similarly, *Escherichia-Shigella* and *Campylobacter* are known to be associated with intestinal inflammation and dysbiosis, and their proliferation often results in adverse effects on growth and overall health in chickens [3, 60].

The predicted metabolic pathway analysis revealed that the LHW-SB3 and HHW-SB3 groups clustered together on d 14, indicating similar metabolic responses to SB despite initial weight differences. The LHW-SB3 group exhibited the highest abundance of microbial pathways involved in the production of gondoic acid, a known antimicrobial agent effective against Gram-negative bacteria [55]. The enrichment of this metabolic pathway, combined with the increased expression of *IL-10* in the LHW-SB3 group indicates the potential for reduced inflammatory responses and the exclusion of Gram-negative bacteria, which are commonly linked to enteric diseases. The HHW-SB3 group exhibited relative enrichment in the bifidum fermentation pathway, which improves gut health through acetate and lactate production [61]. Additionally, the glycine-serine microbial pathway was more abundant in the HHW-SB3 group, indicating increased amino acid synthesis. Glycine is crucial for modern broiler chickens due to its limited endogenous synthesis [62]. Glycine also has anti-inflammatory effects, suppressing transcription factors, free radicals, and cytokine production in macrophages [63], which is beneficial to host health.

In ovo SB may exert different effects than in-feed administration due to the timing and duration of exposure. In ovo SB injection delivers a single, critical dose early in development, likely inducing epigenetic and microbiota changes. These alterations may trigger cascading physiological effects that persist until d 42 post-hatch. Future research should focus on larger-scale trials to validate these findings and explore the underlying epigenetic and microbiota-mediated mechanisms more comprehensively.

## Conclusions

HW had a positive effect on subsequent broiler growth performance and the HHW-control group demonstrated better growth performance and a more favorable gut microbiota characteristics compared to the LHW-control group. Butyrate appeared to exert a more significant effects on LHW chicks at 0.3% inclusion level, likely due to their compromised gut health. This led to significant improvements in intestinal development, strengthened gut barrier function, increased anti-inflammatory cytokine production, and beneficial cecal microbiota characteristics, collectively contributing to enhanced growth performance. The effects of SB were dose dependent, with adverse outcomes observed at higher concentrations (0.5%), impacting performance, the gut microbiota, and the expression of intestinal genes. These results provide insights into optimizing SB use for broilers with varying HW. The potential for targeted intervention is particularly promising for LHW chicks, presenting an opportunity to reduce BW variance among broilers to improve overall flock uniformity.

## Supplementary Information

**Additional file 1:** **Table S1 **Primers used for gene expression analysis via qPCR and a brief description of their main functions. **Fig. S1 **Hatchability of eggs subjected to in ovo injection of different sodium butyrate (SB) doses. The data were analyzed by one-way ANOVA (*n* = 10 repetitions per treatment). The control group received normal saline, while the other groups received SB at 0.1% (SB1), 0.3% (SB3) or 0.5% (SB5). **Fig. S2 **Boxplot showing the pairwise Bray‒Curtis dissimilarity between groups of high (HHW) and low (LHW) hatch weight (HW) chickens on d 14 (**A**) and d 42 (**B**) that had received 3 levels of in ovo sodium butyrate (SB1: 0.1%, SB3: 0.3%, SB5: 0.5%) or 0.9% NaCl (control). Individually sampled chickens were considered to be in experimental units (*n* = 6 birds/group). **Fig. S3 **Heatmap of the top 50 predicted metabolic pathways according to the MetaCyc database in high (HHW) and low (LHW) hatch weight chickens on d 14 (**A**) and d 42 (**B**) that had received 3 levels of sodium butyrate (SB1: 0.1%, SB3: 0.3%, SB5: 0.5%) or 0.9% NaCl (control) in ovo. Red indicates a high relative abundance, while blue indicates a low relative abundance of metabolic pathways.

## Data Availability

The 16S rRNA gene sequencing data can be accessed under Bioproject accession PRJNA1142425 at the NCBI website.

## Abbreviations

ADFI: Average daily feed intake
ADG: Average daily gain
ANOVA: Analysis of variance
BW: Body weight
ED: Embryonic day
FCR: Feed conversion ratio
FDR: False discovery rate
GIT: Gastrointestinal tract
HW: Hatch weight
LDA: Linear discriminate analysis
LEfSe: Linear discriminant analysis effect size
OTU: Operational taxonomic unit
PCoA: Principal coordinate analysis
PERMANOVA: Permutational multivariate analysis of variance
Picrust2: Phylogenetic investigation of communities by reconstruction of unobserved states
SB: Sodium butyrate
SCFA: Short-chain fatty acid
SD: Standard deviation
