# Supplementary material for: In ovo sodium butyrate administration differentially impacts growth performance, intestinal barrier function, immune response, and gut microbiota characteristics in low and high hatch-weight broilers
Source: J Anim Sci Biotechnol. 2024 Dec 7;15:165. doi: 10.1186/s40104-024-01122-4 (PMC11624594; doi:10.1186/s40104-024-01122-4)
Supplement: Supplementary file 1 — Additional file 1: Table S1 Primers used for gene expression analysis via qPCR and a brief description of their main functions. Fig. S1 Hatchability of eggs subjected to in ovo injection of different sodium butyrate (SB) doses. The data were analyzed by one-way ANOVA (n = 10 repetitions per treatment). The control group received normal saline, while the other groups received SB at 0.1% (SB1), 0.3% (SB3) or 0.5% (SB5). Fig. S2 Boxplot showing the pairwise Bray‒Curtis dissimilarity between groups of high (HHW) and low (LHW) hatch weight (HW) chickens on d 14 (A) and d 42 (B) that had received 3 levels of in ovo sodium butyrate (SB1: 0.1%, SB3: 0.3%, SB5: 0.5%) or 0.9% NaCl (control). Individually sampled chickens were considered to be in experimental units (n = 6 birds/group). Fig. S3 Heatmap of the top 50 predicted metabolic pathways according to the MetaCyc database in high (HHW) and low (LHW) hatch weight chickens on d 14 (A) and d 42 (B) that had received 3 levels of sodium butyrate (SB1: 0.1%, SB3: 0.3%, SB5: 0.5%) or 0.9% NaCl (control) in ovo. Red indicates a high relative abundance, while blue indicates a low relative abundance of metabolic pathways. [file 40104_2024_1122_MOESM1_ESM.pdf]

Table S1: Primers used for gene expression analysis via qPCR and a brief description of their main functions.

[1]Gene	Function	Primer sequences (5’ to 3’)	Reference
CLDN1	Barrier integrity and permeability regulation	F: TCTTCATCATTGCAGGTCTGTC<br>R: AACGGGTGTGAAAGGGTCAT	[1]
TJP1	Regulates cellular tight junctions	F: AGGAAGCGATGAATCCCTGTT<br>R: TCACTCAGATGCCAGATCCAA	[1]
MUC6	Produces protective mucus	F: TTCAACATTCAGTTCCGCCG<br>R: TTGATGACACCGACACTCCT	[1]
IL-10	Anti-inflammatory cytokine: Interleukin 10	F: CATGCTGCTGGGCCTGAA<br>R:CGTCTCCTTGATCTGCTTGATG	[1]
IL-1B	Pro-inflammatory cytokine: interleukin 1 beta	F: GGAGGTTTTTGAGCCCGTC<br>R: TCGAAGATGTCGAAGGACTG	[1]
IL-12p40	Pro-inflammatory cytokine: interleukin-12 subunit p40	F: TTGCCGAAGAGCACCAGCCG<br>R: CGGTGTGCTCCAGGTCTTGGG	[1]
ACTB	Reference gene: Actin beta	F:CACAGATCATGTTTGAGACCTT<br>R: CATCACAATACCAGTGGTACG	[1]
G6DPH	Reference gene: Glucose-6-phosphate dehydrogenase	F: CGGGAACCAAATGCACTTCGT<br>R: GGCTGCCGTAGAGGTATGGGA	[1]

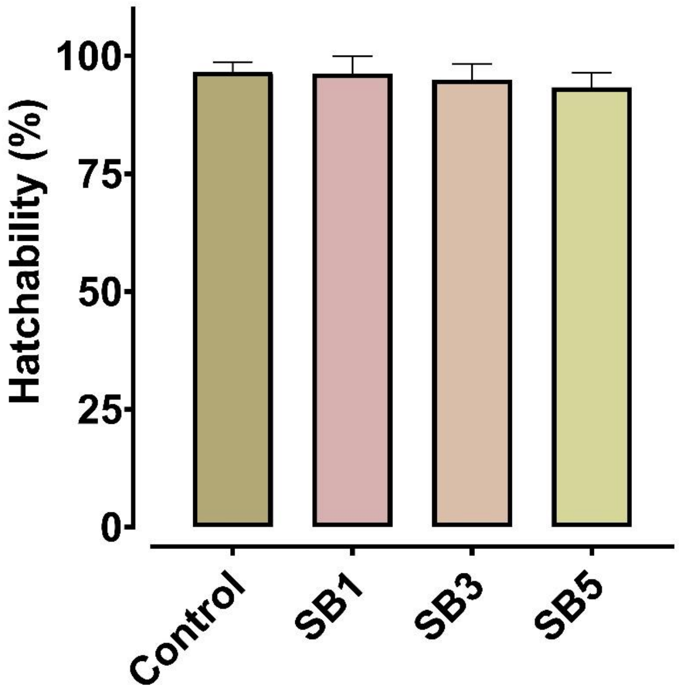

Fig. S1: Hatchability (%) of eggs subjected to in ovo injection of different sodium butyrate (SB) doses. The data were analyzed by one-way ANOVA (*n* = 10 repetitions per treatment). The control group received normal saline, while the other groups received SB at 0.1% (SB1), 0.3% (SB3) or 0.5% (SB5).

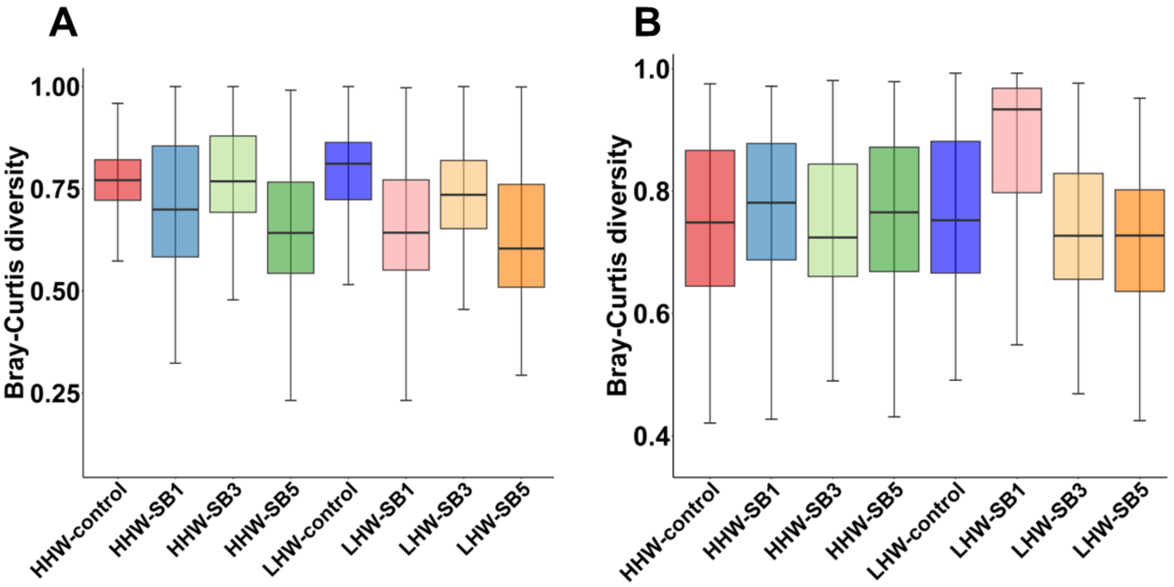

Fig. S2: Boxplot showing the pairwise Bray–Curtis dissimilarity between groups of high (HHW) and low (LHW) hatch weight (HW) chickens on d 14 (A) and d 42 (B) that had received three levels of in ovo sodium butyrate (SB1: 0.1%, SB3: 0.3%, SB5: 0.5%) or 0.9% NaCl (control). Individually sampled chickens were considered to be in experimental units (*n* = 6 birds/group).

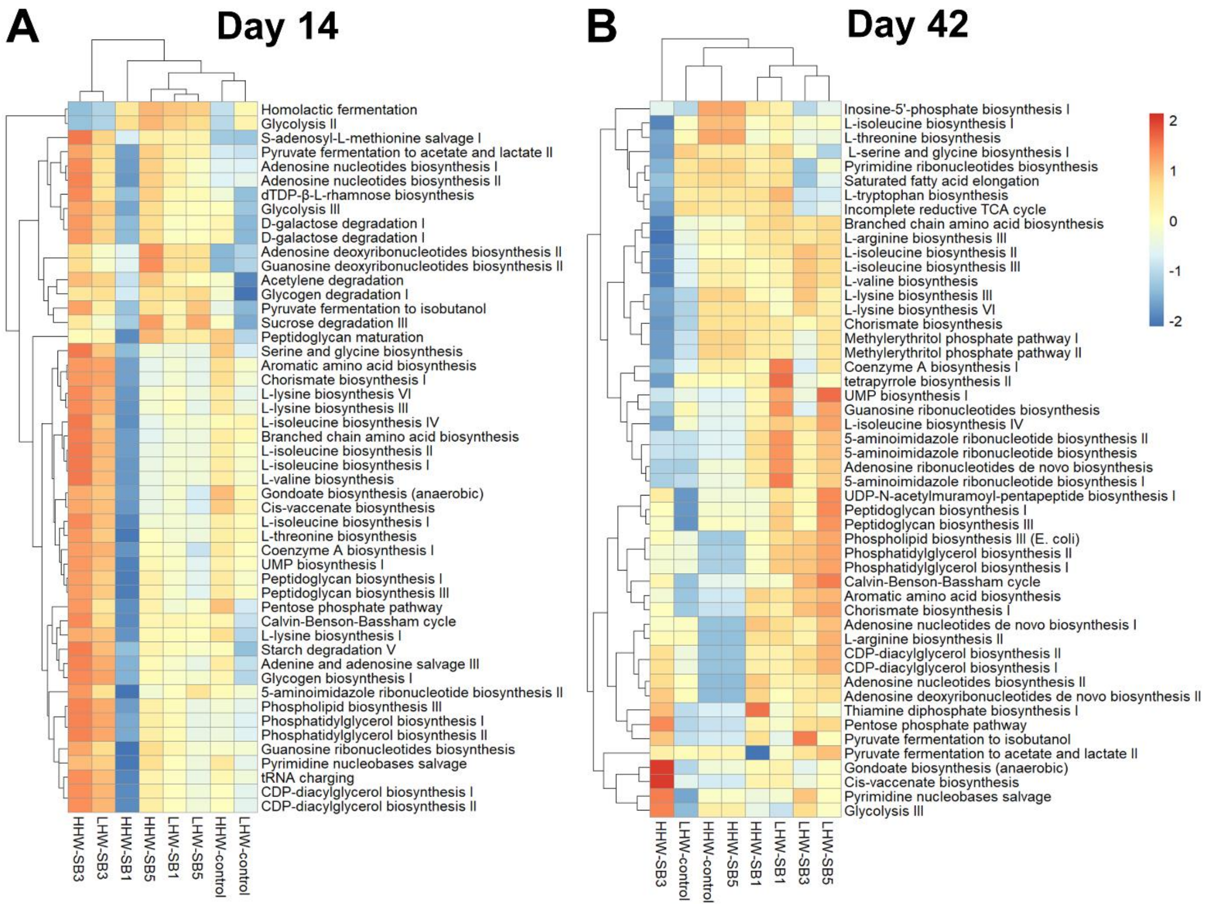

Fig. S3: Heatmap of the top 50 predicted metabolic pathways according to the MetaCyc database in high (HHW) and low (LHW) hatch weight chickens on d 14 **(A)** and d 42 **(B)** that had received three levels of sodium butyrate (SB1: 0.1%, SB3: 0.3%, SB5: 0.5%) or 0.9% NaCl (control) in ovo. Red indicates a high relative abundance, while blue indicates a low relative abundance of metabolic pathways.

References

1. Slawinska A, Dunislawska A, Plowiec A, Radomska M, Lachmanska J, Siwek M, et al. Modulation of microbial communities and mucosal gene expression in chicken intestines after galactooligosaccharides delivery In Ovo. PLoS One. 2019;14:1–23.
